# Hydroxyl Radical Overproduction in the Envelope: an Achilles’ Heel in Peptidoglycan Synthesis

**DOI:** 10.1128/spectrum.01203-21

**Published:** 2022-02-16

**Authors:** Sean Giacomucci, Laura Alvarez, Christopher D. A. Rodrigues, Felipe Cava, Catherine Paradis-Bleau

**Affiliations:** a Département de Microbiologie, Infectiologie et Immunologie, Faculté de Médecine, Université de Montréal, Montréal, Québec, Canada; b Molecular Infection Medicine Sweden, Umea University, Umeå, Sweden; c Umea Center for Microbial Research, Umea University, Umeå, Sweden; d iThree Institute, University of Technology Sydney, Ultimo, New South Wales, Australia; e School of Life Sciences, University of Warwick, Coventry, United Kingdom; Griffith University

**Keywords:** oxidative stress, peptidoglycan synthesis, bacterial envelope biology, reactive oxygen species, hydroxyl radical, Fenton reaction, iron homeostasis, peptidoglycan

## Abstract

While many mechanisms governing bacterial envelope homeostasis have been identified, others remain poorly understood. To decipher these processes, we previously developed an assay in the Gram-negative model Escherichia coli to identify genes involved in maintenance of envelope integrity. One such gene was ElyC, which was shown to be required for envelope integrity and peptidoglycan synthesis at room temperature. ElyC is predicted to be an integral inner membrane protein with a highly conserved domain of unknown function (DUF218). In this study, and stemming from a further characterization of the role of ElyC in maintaining cell envelope integrity, we serendipitously discovered an unappreciated form of oxidative stress in the bacterial envelope. We found that cells lacking ElyC overproduce hydroxyl radicals (HO^•^) in their envelope compartment and that HO^•^ overproduction is directly or indirectly responsible for the peptidoglycan synthesis arrest, cell envelope integrity defects, and cell lysis of the Δ*elyC* mutant. Consistent with these observations, we show that the Δ*elyC* mutant defect is suppressed during anaerobiosis. HO^•^ is known to cause DNA damage but to our knowledge has not been shown to interfere with peptidoglycan synthesis. Thus, our work implicates oxidative stress as an important stressor in the bacterial cell envelope and opens the door to future studies deciphering the mechanisms that render peptidoglycan synthesis sensitive to oxidative stress.

**IMPORTANCE** Oxidative stress is caused by the production and excessive accumulation of oxygen reactive species. In bacterial cells, oxidative stress mediated by hydroxyl radicals is typically associated with DNA damage in the cytoplasm. Here, we reveal the existence of a pathway for oxidative stress in the envelope of Gram-negative bacteria. Stemming from the characterization of a poorly characterized gene, we found that HO^•^ overproduction specifically in the envelope compartment causes inhibition of peptidoglycan synthesis and eventually bacterial cell lysis.

## INTRODUCTION

The creation of new antibacterial molecules is essential to respond to the global problem of antibiotic resistance. As such, new bacterial targets must be found. Many antibiotics targeting Gram-positive bacteria are inefficient against Gram-negative bacteria, primarily due to the presence of an outer membrane in the Gram-negatives (1). The peptidoglycan (PG) layer is an essential element of the bacterial envelope. It is required for envelope integrity, defines the cell shape, and is essential for coping with changes in osmotic pressure (2). Bacteria need to constantly produce and remodel their PG to preserve cellular integrity and adaptability and allow growth and division. Prats and de Pedro (3) demonstrated that Escherichia coli cells can grow normally and exhibit no detectable morphological defects with approximately 50% less PG per cell, compared to wild type (WT). Several studies agree that inhibition of PG synthesis ultimately leads to cell lysis due to the imbalance between synthetic and degrading machineries (4–7). PG is located in the periplasmic compartment between the inner and outer membranes in Gram-negative bacteria. It is a complex polymer composed of chains made of two alternating sugars, cross-linked by short peptides. Sugar moieties are *N*-acetylglucosamine (GlcNAc), and *N*-acetylmuramic acid (MurNAc) that is bound to a peptide moiety (8, 9). PG precursors are produced in the cytoplasm and assembled on the lipid carrier undecaprenyl phosphate (C55-P) in the inner leaflet of the inner membrane. This precursor, named lipid II, is then flipped to the outer leaflet of the inner membrane and added to preexisting PG chains by PG synthases (10).

The molecular pathways governing Gram-negative bacteria envelope biogenesis, dynamics, and homeostasis are highly complex and are still not well understood. To discover processes governing envelope biology, we previously developed an assay to identify all the nonessential genes required to maintain cell envelope integrity in the Gram-negative model system E. coli (11). This assay involves the yellow-colored LacZ substrate chlorophenol red-β-d-galactopyranoside (CPRG), which cannot cross healthy cell envelopes. The cytoplasmic LacZ enzyme thus does not have access to this envelope-impermeable substrate unless the integrity of the envelope is comprised. Envelope integrity defects lead to CPRG being hydrolyzed by LacZ into the pink-colored chlorophenol red (CPR) product for a positive readout in the assay (a CPRG^+^ phenotype) (11). This screen identified over a hundred genes of unknown function as being required for Gram-negative envelope integrity. The Δ*elyC* (formerly Δ*ycbC*) mutant had one of the strongest CPRG^+^ phenotypes. ElyC is predicted to be an inner membrane protein with a highly conserved domain of unknown function, DUF218 (12). This domain has been reported in all superkingdoms; in bacteria, the DUF218 domain can be found in almost all bacterial phyla, including in strictly anaerobic species (12, 13). In bacteria, DUF218 domain-containing proteins from E. coli and Salmonella enterica serovar Typhimurium have been implicated in vancomycin resistance, envelope integrity, and anaerobic respiration (14–17).

We previously demonstrated that cells lacking *elyC* display a lysis phenotype caused by a PG synthesis arrest at room temperature. The cell lysis phenotype could be suppressed by overexpressing genes involved in PG synthesis that encode MurA, which performs the first committed step of PG precursor synthesis in the cytoplasm, the periplasmic PG synthase PBP1B, and UppS, which is responsible for the production of the lipid carrier C55-P (11). The Δ*elyC* mutant cell lysis phenotype was also suppressed by deleting certain genes involved in the biogenesis of enterobacterial common antigen (ECA), a surface polysaccharide whose synthesis also involves C55-P (10). These observations led to the hypothesis that the Δ*elyC* cell lysis phenotype is suppressed by increasing the availability of the lipid carrier C55-P (11). Furthermore, we found that the defective phenotypes of Δ*elyC* mutant cells can be suppressed by the overexpression of *dsbG*. Interestingly, periplasmic DsbG suppressed the mutant defects independently of its reductase activity, instead suppressing the defects by its chaperone activity. The periplasmic chaperone Spy could also suppress the mutant defects, but cytoplasmic chaperones could not. Importantly, we detected protein aggregation in the periplasm of Δ*elyC* cells, which indicates a perturbation of the biology of this compartment in the absence of ElyC (18).

Interestingly, in this work, while attempting to decipher the mechanistic origin of the PG synthesis arrest in Δ*elyC*, we discovered that the mutant lysis phenotype is dependent on aeration. This observation leads us to hypothesize that the PG synthesis arrest in Δ*elyC* is related to oxidative stress. During aerobic respiration, cells produce reactive oxygen species (ROS) that can alter proteins, DNA, RNA, and even lipids (19, 20). ROS are produced from molecular oxygen (O_2_) and include the following: superoxide radical (O_2_^•−^), hydrogen peroxide (H_2_O_2_) and hydroxyl radical (HO^•^). The most damaging ROS is HO^•^, and it is produced from H_2_O_2_ in the presence of free ferrous iron (Fe^2+^) through the Fenton reaction (21, 22). While O_2_^•−^ and H_2_O_2_ oxidizing efficiency are respectively lowered by a negative charge and a stable oxygen-oxygen bond, HO^•^ is highly unstable, has no charge, and can react with almost all molecules within the bacterial cell (23). HO^•^ has an extremely high reactivity with a half-life of 10^−9^ s, which makes it the most hazardous ROS (23, 24). Although iron is essential for bacteria, labile iron pools in bacteria are tightly controlled due to its high reactivity with ROS, which makes it toxic. The import, export, redox state, storage, scavenging, and release of iron are regulated to avoid excess of free iron within bacterial cells and to ensure that iron is available for incorporation into proteins (23, 25–29). HO^•^ radicals are known to be produced in the cytoplasm by the release of iron from its scavengers (Dps or ferritin-like proteins). As iron has a high affinity for nucleic acids, DNA is extremely susceptible to oxidation mediated by Fenton-generated HO^•^, which leads to damaged bases and DNA breaks. Chromosomal breaks activate the SOS response, which delays cell division and thereby causes bacterial filamentation (bacteria continue growing but are unable to divide) (19, 23, 30). Although the *ΔelyC* mutant phenotype differs from what is generally observed in cases of HO^•^ overproduction, we decided to investigate its susceptibility to O_2_ and test the hypothesis that inhibition of PG synthesis and cell lysis in *ΔelyC* cells are related to ROS production.

In this study, we show that *ΔelyC* mutant cells overproduce HO^•^. The inhibition of the Fenton reaction or anaerobic growth conditions restored the *ΔelyC* mutant phenotypes to WT levels, including PG synthesis. We also found that Δ*elyC* mutant cells are highly susceptible to chromate-mediated oxidative stress. Importantly, we show that HO^•^ production in Δ*elyC* mutant cells does not damage DNA, and thus HO^•^ is not likely produced in the cytoplasm but in the envelope, a result that is consistent with our previous work showing protein aggregation in the periplasm of *ΔelyC* mutant cells (18). To our knowledge, HO^•^ has not previously been described to have an impact on PG synthesis. Even though this phenotype might occur in a specific genetic context and under specific conditions, we have identified an unappreciated weakness in PG biosynthetic biology, involving a likely molecular cascade leading to HO^•^ overproduction in the bacterial envelope.

## RESULTS

### The Δ*elyC* mutant envelope defect and terminal lysis phenotype require medium oxygenation.

**FIG 1:**
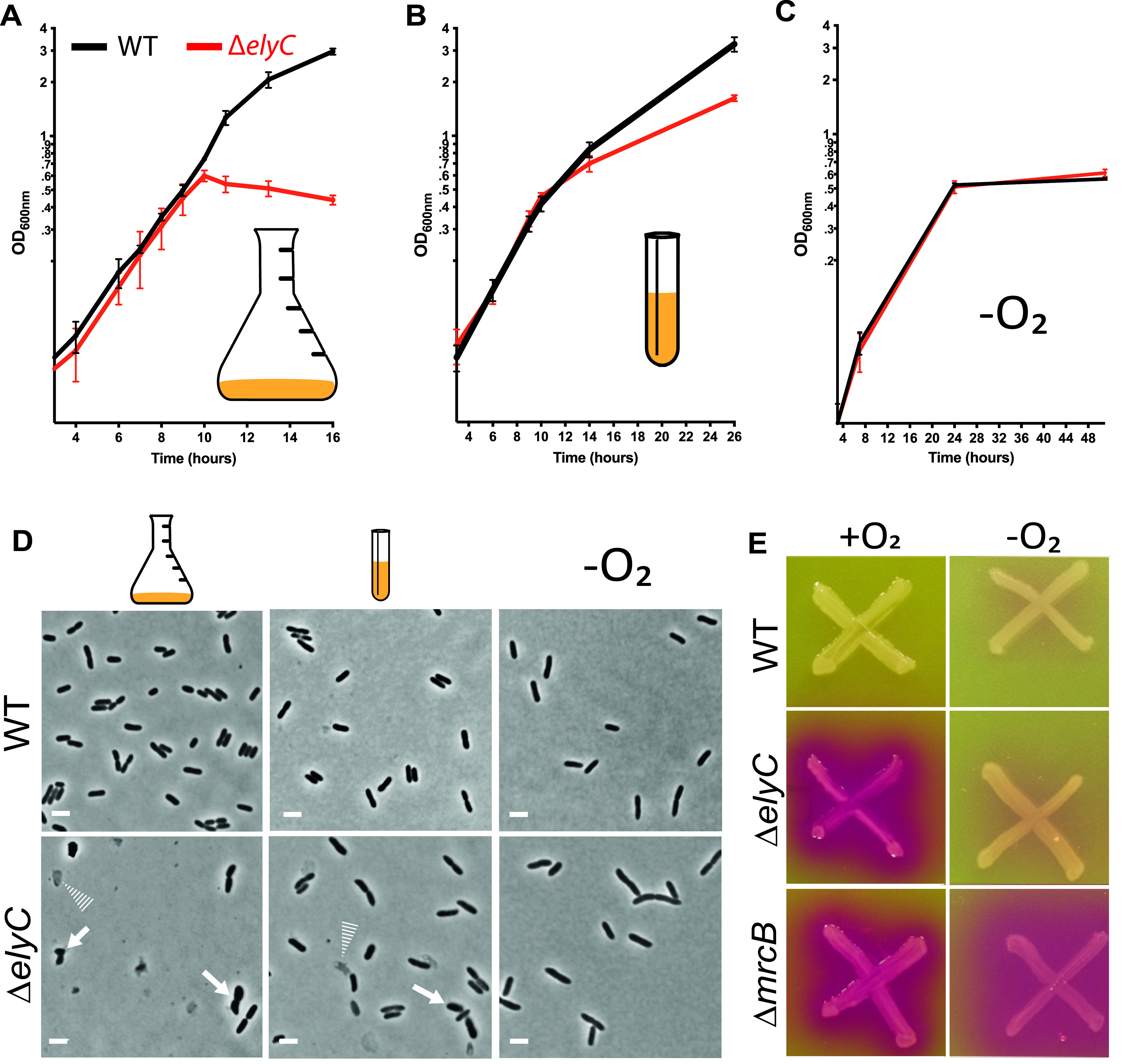
The envelope defect and lysis phenotype of Δ*elyC* cells depend on medium aeration. (A to C) Growth curves of wild-type (WT) (black curves) and Δ*elyC* (red curves) cells at 21°C. The values represented correspond to the mean of the optical density at 600 nm (OD_600nm_) measurements of at least three biological replicates ± standard deviation (SD). The cultures were grown as follows: 25 mL in 250-mL Erlenmeyer flasks (A); 5 mL in narrow 10-mL culture tubes (B); and in anaerobic conditions (−O_2_) (C). Shown is a representative data set from experiments performed in biological triplicates. (D) Phase contrast microscopy imaging at 100× showing cell morphology observed after 12 h of culture in flasks and culture tubes or after 24 h for anaerobic growth. The cells were grown under the same conditions as in panels A to C. The conditions are indicated by pictograms; respectively from left to right: Erlenmeyer flask, culture tube, and anaerobic conditions. Solid arrows point to misshaped, bulging, or lysing cells, and dotted arrows point bacterial ghosts. Bar = 3 μm. (E) Chlorophenol red-β-d-galactopyranoside (CPRG) assay plate for the WT, Δ*elyC*, and Δ*mrcB* strains incubated at 21°C in aerobic (+O_2_) and anaerobic conditions (−O_2_).

Our previous study demonstrated that ElyC is important for bacterial cell envelope integrity and more specifically for proper PG biogenesis (11). In an effort to discover how ElyC functions in PG biogenesis, we investigated the Δ*elyC* phenotypes under different conditions. Interestingly, we noticed that the growth dynamics of Δ*elyC* cells differed depending on the specific glassware used for cultivation and the volume of liquid media they contained. We previously showed that when a subculture of Δ*elyC* cells grown at 37°C was transferred to 21°C, Δ*elyC* cells grew as well as WT cells until an optical density at 600 nm (OD_600nm_) of approximately 0.6 but then stopped growing and lysed (11). When we cultured Δ*elyC* cells at 21°C in 25 mL of liquid medium in 250-mL Erlenmeyer flasks (one-tenth of the total glassware volume), under agitation at 250 rpm, mutant cells grew as well as WT cells until an OD_600nm_ of 0.5 to 0.6 and then lysed (Fig. 1A and D). In contrast, when Δ*elyC* cells were cultured at 21°C in 5 mL of liquid media in narrow 10-mL culture tubes (one-half of the total glassware volume), under agitation, we did not observe the complete cell lysis phenotype but only a reduction in growth rate in comparison to WT cells (Fig. 1B). When observed under the microscope, some Δ*elyC* cells grown in the narrow tubes, but not all, displayed signs of envelope defects: some cells had lysed, while others appeared as bacterial ghosts (empty cell envelopes) (31) (Fig. 1D and Fig. S2 and S3). The severity of the lysis phenotype of the Δ*elyC* cells thus varied depending on growth conditions. We reasoned that the main difference in the culture conditions was the medium oxygenation from the agitation. Indeed, the potential for medium aeration and thus oxygenation is much higher in the flask condition than in the culture tube condition. We reasoned that increasing medium aeration by growing Δ*elyC* mutant cells in large flasks with low volume of liquid media preempted the terminal phenotype of lysis, whereas growing mutant cells in small, half-full culture tubes reduced the Δ*elyC* defects. We conclude that the lysis phenotype of Δ*elyC* mutant cells appears to be dependent on medium aeration and hypothesized that the envelope defect of the Δ*elyC* mutant requires the presence of molecular oxygen.

We reasoned that if our hypothesis is correct, growing Δ*elyC* cells in the absence of oxygen would completely alleviate all defective phenotypes. Indeed, we observed no differences in Δ*elyC* and WT growth kinetics (Fig. 1C) and cellular morphologies (Fig. 1D and Fig. S3) under anaerobic conditions. Furthermore, Δ*elyC* cells did not display a CPRG^+^ phenotype, indicative of envelope defects, when the assay was performed anaerobically (Fig. 1E), indicating that Δ*elyC* cells maintain envelope integrity in the absence of oxygen. Importantly, the Δ*mrcB* mutant (a gene implicated in PG synthesis/remodeling and used as an envelope-defective control strain in the assay) maintained its CPRG^+^ phenotype without oxygen exposure (Fig. 1E).

Altogether, these results show that an increase in medium aeration exacerbates the Δ*elyC* defects, while anaerobic conditions completely suppress them. This establishes that molecular oxygen is involved in the development of envelope defects in Δ*elyC* cells.

### Δ*elyC* mutant cells produce high levels of hydroxyl radicals (HO^•^) at 21°C.

**FIG 2:**
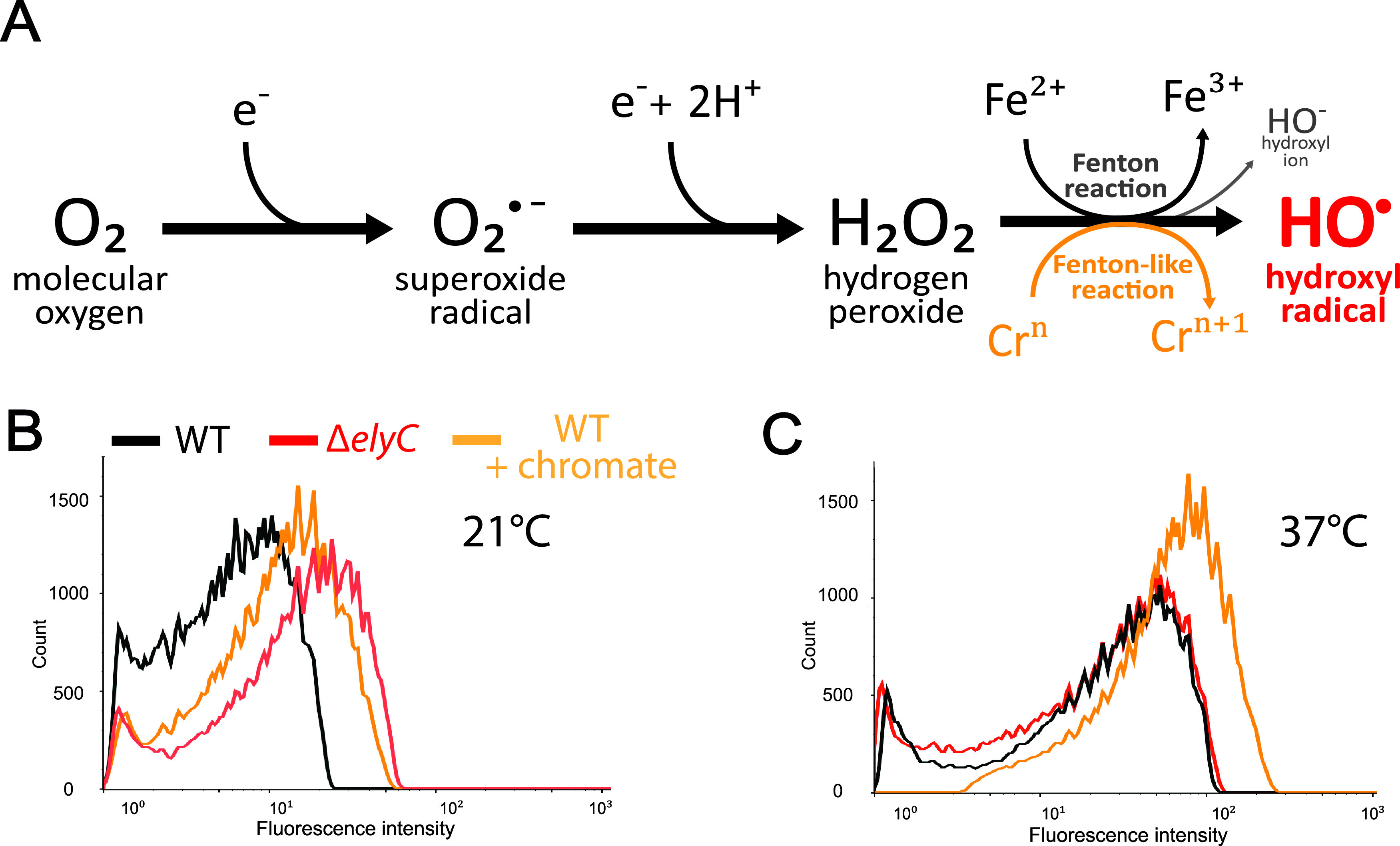
Δ*elyC* mutant cells produce high levels of HO• at 21°C. (A) Simplified molecular scheme representing the production of reactive oxygen species (ROS), superoxide radical (O_2_^•−^), hydrogen peroxide (H_2_O_2_), and hydroxyl radical (HO^•^). Successive univalent electron (e^−^) leakages to oxygen form O_2_^•−^ and H_2_O_2_ in the presence of protons and free ferrous iron (Fe^2+^). HO^•^ are generated from H_2_O_2_ in the presence of Fe^2+^ through the Fenton reaction, releasing a hydroxyl ion (HO^−^) and ferric iron (Fe^3+^). Chromium at its III or V (Cr*^n^*) oxidation state induces HO^•^ production through a Fenton-like reaction (35). (B, C) Flow cytometry histograms showing fluorescein detection at 515 nm (±50 nm) by the number of events from the hydroxyphenyl fluorescein (HPF) probe used to evaluate HO^•^ levels at 21°C (B) and 37°C (C). WT cells (black curves), Δ*elyC* cells (red curves), and WT cells were grown with 120 μM potassium chromate as a positive control on the assay for increased HO^•^ production (orange curves).

Since oxygen is the most potent electron acceptor, oxidative stress is inevitably present under aerobic conditions. We hypothesized that oxidative stress is the underlying cause of the oxygen dependency of the envelope defect in Δ*elyC* and that mutant cells would contain higher, detrimental amounts of ROS. As HO^•^ is by far the most reactive and hazardous ROS and as O_2_^•−^ and H_2_O_2_ production can result in HO^•^ formation (23, 27), we identified HO^•^ as the best potential candidate for triggering such a severe phenotype as cell lysis. We measured HO^•^ by flow cytometry using the hydroxyphenyl fluorescein (HPF) probe. Fluorescence develops from HPF when it reacts with HO^•^, leading to HPF oxidation and its cleavage into fluorescein (32, 33). We note that while HPF oxidation has been shown to be a measure of HO^•^ production, it may also report on peroxynitrite, a reactive nitrogen species (33). However, based on the results reported here, we believe that HPF oxidation is reporting on HO^•^ levels (see the Discussion). As a positive control in the assay, we used potassium chromate (K_2_CrO_4_), which is known to induce the production of HO^•^ through a Fenton-like reaction (34–38) (Fig. 2). We compared HO^•^ production in Δ*elyC* and WT cells grown in flasks at 21 and 37°C until OD_600nm_ of 0.35, about one generation before lysis for Δ*elyC* cells (Fig. 1A).

The addition of potassium chromate increased fluorescence emission 2-fold in WT cells at 21°C and 3-fold at 37°C, indicating that the HO^•^ detection assay was functional (Fig. 2B and Fig. S6A and B). Fluorescence intensity was overall higher at 37°C than at 21°C. At 21°C, Δ*elyC* cells produced a fluorescence signal that was 3-fold higher than WT cells and 1.5-fold higher than WT cells treated with potassium chromate (Fig. 2B and Fig. S6A). Conversely, Δ*elyC* and WT cells had an identical fluorescence signal at 37°C (Fig. 2C and Fig. S6B). As the terminal lysis phenotype of Δ*elyC* cells occurred in flasks at 21°C but not at 37°C (11), these data indicate that mutant cells produce elevated levels of HO^•^ specifically in growth conditions leading to the phenotype of lysis. As a fluorescein-based fluorescent probe, like HPF, could be sensitive to the pH of the solution (39), we measured the influence of K_2_CrO_4_ on the pH of LB medium (Table S2). We observed that K_2_CrO_4_ at our working concentration had no effect on the medium pH, indicating that the increase in HPF signal was due to HO^•^ overproduction.

### Excess production of HO^•^ by the iron-dependent Fenton reaction triggers Δ*elyC* cell lysis through PG synthesis inhibition.

**FIG 3:**
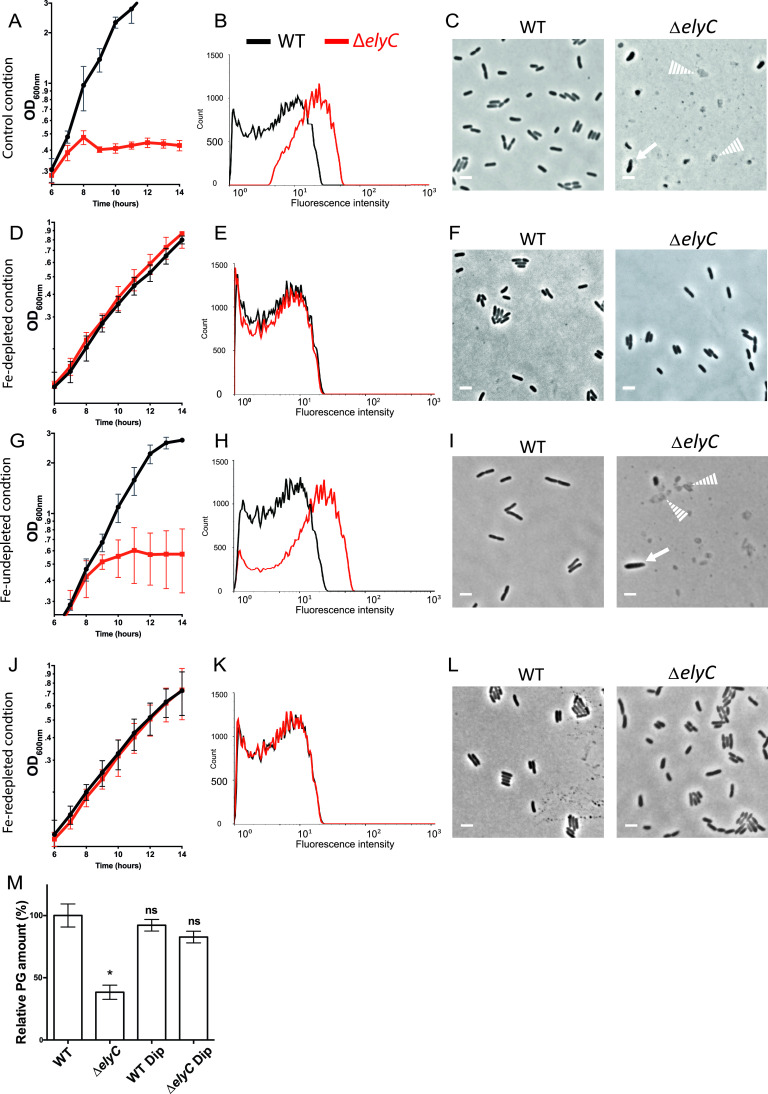
HO•-generating free iron is necessary for Δ*elyC* peptidoglycan (PG) synthesis inhibition and cell lysis at 21°C. (A, D, G, J) Growth curves of WT (black curves) and Δ*elyC* (red curves) cells grown in flasks. The values represented correspond to the mean of OD_600nm_ measurements of at least three biological replicates ± SD. (B, E, H, K) Flow cytometry histograms reporting HPF probe fluorescence signal to measure HO^•^ levels in WT (black curves) and Δ*elyC* (red curves) cells grown until OD_600nm_ = 0.35. (C, F, I, L) Phase contrast microscopy imaging at 100× showing cell morphology observed after 12 h of culture. Solid arrows point to lysing or misshaped cells. Dotted arrows point to bacterial ghosts. Bar = 3 μm. Specific growth conditions are indicated on the left of each serial figure line. (A to C) Control conditions. (D to F) Fe-depleted conditions (cultures supplemented with 375 μM 2,2′-dipyridyl). (G to I) Fe-undepleted conditions (cultures supplemented with 375 μM 2,2′-dipyridyl and 100 μM FeSO_4_). (J to L) Fe-redepleted conditions (cultures supplemented with 600 μM 2,2′-dipyridyl and 100 μM FeSO_4_). (M) Bar graph showing the relative PG amount of WT and Δ*elyC* cells grown with or without 375 μM 2,2′-dipyridyl (Dip) until OD_600nm_ = 0.5. The total PG amount of WT cells was defined as the 100% reference point. The data are expressed as means ± standard error of the mean (SEM). The asterisk indicates a significant difference from the reference condition (*P < *0.05). ns, not significant.

We hypothesized that the elevated HO^•^ production in Δ*elyC* cells induces bacterial lysis through PG synthesis inhibition. Since HO^•^ formation requires free iron as an electron donor to feed the Fenton reaction (23) (Fig. 2A), we reasoned that chelating iron would inhibit the Fenton reaction and consequently decrease HO^•^ production, thereby suppressing the Δ*elyC* cell lysis phenotype. We used the iron chelator 2,2′-dipyridyl, which freely diffuses into bacterial membranes (40, 41). We titrated 2,2′-dipyridyl to define the precise minimum concentration required to rescue the mutant from lysis. The addition of 375 μM 2,2′-dipyridyl was sufficient to restore Δ*elyC* growth kinetics to WT levels (Fig. 3D). As 2,2′-dipyridyl chelates cellular iron, an essential cofactor in many metabolic processes (26), bacterial growth was consistently slower for both strains in the Fe-depleted condition (Fig. 3A and D). As expected, addition of 375 μM 2,2′-dipyridyl resulted in Δ*elyC* cells producing the same amount of HFP signal as WT cells (Fig. 3B and E). Thus, limiting the HO^•^ production in Δ*elyC* cells by inhibiting the Fenton reaction with 2,2′-dipyridyl alleviated the lysis, growth, and morphological defects of the mutant at 21°C (Fig. 3A to F and Fig. S2 and S3). Furthermore, when 2,2′-dipyridyl was added to Δ*elyC* cell cultures after 6 h of growth at 21°C, about 2 h before lysis, it successfully rescued the mutant terminal phenotype, and no lysis was observed (Fig. S4). These results support our hypothesis that the high level of HO^•^ produced in Δ*elyC* cells triggers bacterial lysis. These results are also consistent with free ferrous iron generating the toxic levels of HO^•^ in Δ*elyC* cells through the Fenton reaction.

To test this model further, we tested whether addition of free ferrous iron to cultures of Δ*elyC* cells containing 2,2′-dipyridyl would bring back the toxic levels of HO^•^. Indeed, we found that 100 μM ferrous iron in the form of FeSO_4_ led to the generation of native levels of HO^•^ in Δ*elyC* cells and reproduced the terminal lysis phenotype at 21°C (Fig. 3G to I and Fig. S6A). Thus, the addition of ferrous iron reverts the chelating effect of 2,2′-dipyridyl and reestablishes the excess HO^•^ level in Δ*elyC* cells, along with lysis. To further confirm that the excess production of detrimental HO^•^ by the iron-dependent Fenton reaction in Δ*elyC* cells promotes bacterial lysis, we optimized a third condition in which the concentration of 2,2′-dipyridyl was raised at 600 μM to chelate the additional iron introduced to the cultures in the Fe-undepleted condition, therefore creating the Fe-redepleted condition. The production of HO^•^ in Δ*elyC* cells was then reduced to the WT level (Fig. 3K). As expected, the higher iron chelator concentration reversed the lethal lysis phenotype of Δ*elyC* cells, with the mutant mimicking WT growth and cellular morphology (Fig. 3J and L and Fig. S2 and S3).

We have previously demonstrated that PG synthesis is blocked in Δ*elyC* cells grown in flasks at 21°C (11). Next, we aimed to determine whether HO^•^ overproduction was responsible for the PG synthesis defect in the Δ*elyC* mutant. To do so, we purified PG from WT and Δ*elyC* cells grown with and without 375 μM 2,2′-dipyridyl until OD_600nm_ of 0.5, before the first signs of cell lysis in mutant cells. Quantification of PG material showed that Δ*elyC* cells contained significantly less (about one-third) of the PG material retrieved in WT cells when grown without 2,2′-dipyridyl (Fig. 3M). Interestingly, no significant difference in PG material was observed between Δ*elyC* and WT cells when grown with the iron chelator, suggesting that PG synthesis is restored in the Δ*elyC* mutant by decreasing the HO^•^ levels back to WT levels. Furthermore, the analysis of the PG profiles revealed that despite some differences in relative PG amounts, the structure and composition of PG was almost identical in all the samples analyzed (Fig. S8A to C). These results indicate that the excess level of HO^•^ produced by the iron-dependent Fenton reaction in Δ*elyC* cells triggers a block to PG synthesis leading to cell lysis.

### HO^•^ overproduction most likely occurs in the envelope of Δ*elyC* cells.

HO^•^-mediated oxidative stress is known to induce a filamentation phenotype (23, 42). As the main pool of ferrous iron in bacterial cells is in the cytoplasm and nucleic acids have a high affinity for ferrous iron, the Fenton reaction predominantly occurs near DNA (23). HO^•^ are nonselective and avid oxidants that quickly react with molecules in their close vicinity; they are thus unlikely to diffuse from their origin of production (19, 24, 43). Generated HO^•^ can cause DNA damage such as DNA strand breaks, which activates the SOS stress response (44), inducing the production of the FtsZ inhibitor SulA (also known as *sfiA*), which blocks cell division (45). Bacteria then grow without dividing and form longer cells as a coping mechanism to allow the completion of DNA repair and replication before cell division resumes (19). Since the excess level of HO^•^ produced by the Fenton reaction in Δ*elyC* cells induces a lytic phenotype through PG synthesis inhibition rather than a filamentation phenotype through cell division inhibition, we reasoned that HO^•^ production in Δ*elyC* cells occurs differently than what has been typically described for bacteria. We hypothesized that in Δ*elyC* cells, toxic HO^•^ are not generated in the cytoplasmic compartment where DNA is located but rather in the envelope compartment where PG is located.

**FIG 4:**
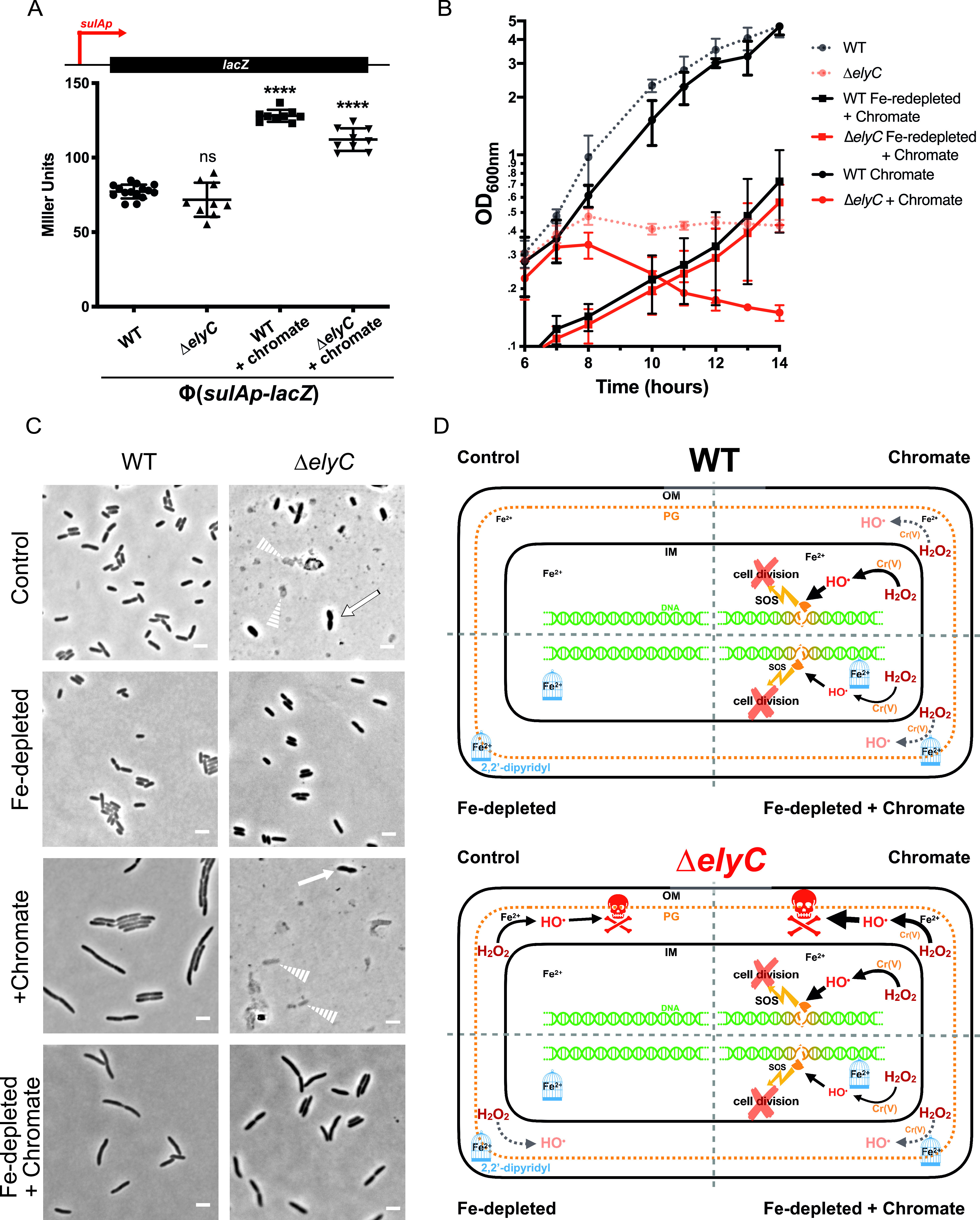
The Δ*elyC* mutant does not show elevated background SOS levels. (A) Expression levels of the SOS-regulated *sulA* promoter, reported by LacZ β-galactosidase activity (measured in Miller units) in WT and Δ*elyC* (EM9)-derived strains: SG24 and SG25, respectively. SG24 (MG1655, Δ(*lacI*-*lacZp*)Φ(Kan^R^*-sulAp*-*lacZ*)) and SG25 (EM9, Δ(*lacI*-*lacZp*)Φ(Kan^R^*-sulAp*-*lacZ*)). The values represented correspond to the mean of OD_600nm_ measurements of at least three biological replicates ± SD. Promoter fusion is represented on top. The cells were grown under control conditions and with potassium chromate (chromate) at 125 μM as a positive control for SOS response activation and was added upon culture set-up. The cells were harvested at OD_600nm_ = 0.35, except for chromate-treated Δ*elyC* cells, which were collected at OD_600nm_ = 0.15. Asterisks indicate a significant difference for chromate-treated cells compared to control WT cells (*P < *0.05). (B) Growth curves of WT (black curves) and Δ*elyC* (red curves) cells. The cells were grown in control condition (dotted faint lines) or with 125 μM chromate (full lines) added after 6 h of incubation (*T* + 6 h). The cells were also grown without (black square) or with (red square) 375 μM 2,2′-dipyridyl at growth initiation (Fe-depleted; see Materials and Methods). (C) Phase contrast microscopy imaging at 100× of WT and Δ*elyC* cells from growth curves in B harvested after 14 h of growth. Solid arrows point to lysing or misshaped cells. Dotted arrows point to bacterial ghosts. Bar = 3 μm. Chromate or 2,2′-dipyridyl were added at growth initiation. (D) Model representing the impact of oxidative stress in WT and Δ*elyC* cells in control, Fe-depleted, chromate, and Fe-depleted + chromate conditions. The skull represents lethal PG damages. Blue cages represent 375 μM 2,2′-dipyridyl chelating labile pool of free ferrous iron (Fe^2+^). Cr(V) represents chromium salt at his fifth oxidation state produced from oxidation of chromate (Cr(VI)) occurring in cells (it could also be represented as Cr(III)). Lightning symbols represent SOS response activation (SOS). Under control conditions, the overproduction of HO^•^ in the envelope of Δ*elyC* cells through the Fenton reaction induces lethal PG defects. In the Fe-depleted condition, HO^•^ overproduction is inhibited by iron chelation, relieving the Δ*elyC* mutant from the PG defect and lysis. When chromate is added to Δ*elyC* mutant cells, HO^•^ produced from Fenton and Fenton-like reactions adds up in the envelope, causing more damage than under control conditions and accelerating the lysis phenotype. In the cytoplasm of WT and Δ*elyC* cells, HO^•^ generated by the Fenton-like reaction induced by chromate causes DNA damages leading to SOS response activation and cell division inhibition. Adding chromate in the Fe-depleted condition leads to cell division inhibition in Δ*elyC* cells. As the Fenton reaction is inhibited by the iron chelator, the native production of high levels of toxic HO^•^ in the envelope of Δ*elyC* cells is suppressed. The Δ*elyC* cells then phenocopy WT cells in reaction to chromate treatment. OM, outer membrane; PG, peptidoglycan; IM, inner membrane.

To test this hypothesis, we studied SOS response activation in Δ*elyC* and WT cells by measuring expression from the *sulA* promoter (*sulAp*). Using the Lambda Red homologous recombination system (46), we substituted the chromosomal *sulA* gene with the *lacZ* reporter gene to create a *sulAp*-*lacZ* transcriptional fusion in Δ*elyC* and WT cells that contained a deletion of their native *lac* operon. We then measured the expression levels from *sulAp* by quantifying LacZ activity with the colorimetric β-galactosidase assay in cells grown in flasks at 21°C until OD_600nm_ of 0.35, about one generation before lysis in Δ*elyC* cells. WT and Δ*elyC* cells displayed the same levels of reporter β-galactosidase activity (Fig. 4A). This result suggests that although the Δ*elyC* mutant produces high levels of HO^•^, the SOS response is not activated in its cytoplasm (Fig. 2B and 4A).

To ensure that the *sulAp*-*lacZ* transcriptional fusion was a functional reporter system for the SOS response, we used potassium chromate as a HO^•^ generator to induce SOS stress. Potassium chromate is a chromium salt in its VI oxidation state, which is known to access the periplasm before entering the cytoplasm through sulfate transporters in the inner membrane (47). Chromium can be reduced to its more reactive forms, mainly at oxidation states V and III, to drive HO^•^ production through a Fenton-like reaction in all bacterial compartments (Fig. 2A) (35, 48). Interestingly, adding potassium chromate to the cultures at growth initiation did not alter the growth kinetic of WT cells but was severely detrimental for Δ*elyC* cells. When chromate was added to the mutant at growth initiation, it caused a more rapid and drastic lysis at an OD_600nm_ of ∼0.25, more than one generation earlier than for untreated cells (Fig. S5). When added to the mutant culture 6 h after growth initiation, chromate is also causing a more rapid and drastic lysis at an OD_600nm_ of ∼0.35, about one generation earlier than for untreated cells (Fig. 4B and Fig. S5). This is consistent with the data presented in Fig. 3, showing that high levels of HO^•^ block PG synthesis, leading to bacterial cell lysis.

Since Δ*elyC* cells lysed quite earlier after addition of potassium chromate, in order to test whether they can induce the SOS response, we added potassium chromate to the mutant cultures at time zero but collected the sample at an early OD. This allowed the recovery of a sufficient amount of mutant cells to quantify LacZ activity with the colorimetric β-galactosidase assay. The growth kinetic of WT cells was not affected by potassium chromate (Fig. 4B), and cells were collected at an OD_600nm_ of 0.35 as described above for untreated cells. WT cells grown with potassium chromate had a 2-fold increase in β-galactosidase activity (Fig. 4A), confirming that potassium chromate induced the SOS response and that the reporter fusion was functional. The addition of potassium chromate to the Δ*elyC* culture at growth initiation (T0) or at the 6-h time point also exacerbated the mutant phenotype as cell lysis occurred respectively at OD_600nm_ levels of 0.35 and 0.25, about one and almost two generation earlier than for untreated mutant cells (Fig. 4B and Fig. S5). To this end, we harvested Δ*elyC* cells at an OD_600nm_ of 0.15, before the premature lysis, and observed a similar 2-fold increase in β-galactosidase activity, as in WT cells (Fig. 4A). This result supports the idea that toxic HO^•^ radicals that are typically present in Δ*elyC* cells at 21°C are not generated in the cytoplasm and thus do not generate an SOS response. Microscopy images confirmed that while potassium chromate induced the SOS filamentation phenotype in WT cells, it triggered an acute lysis phenotype in Δ*elyC* cells (Fig. 4C). Altogether, these results suggest that potassium chromate-induced HO^•^ production adds to the already elevated and toxic HO^•^ pool in Δ*elyC* mutant cells, thus worsening the terminal lysis phenotype.

Next, we hypothesized that alleviating the excess of HO^•^ in Δ*elyC* cells would lead to filamentation instead of lysis upon potassium chromate treatment. We thus added 375 μM 2,2′-dipyridyl to the cultures, in addition to potassium chromate, to study the resulting phenotypes. Indeed, when the iron chelator was added (reducing the levels of HO^•^ in Δ*elyC* cells to WT levels), potassium chromate caused a moderate filamentation phenotype in Δ*elyC* cells that phenocopied WT cells (Fig. 4B to D). The filamentation phenotype was less prominent with 2,2′-dipyridyl than in its absence (Fig. 4C). This is probably due to the growth rate inhibition caused by 2,2′-dipyridyl iron chelation and the reduced synergistic effect of iron and potassium chromate in the production of HO^•^. Altogether, these data support our hypothesis that HO^•^ overproduction occurs in the envelope compartment of Δ*elyC* cells, thereby weakening the envelope structure.

### Free extracytoplasmic iron is necessary for the elevated HO^•^ production in Δ*elyC* cells.

Since ROS appeared to be produced in the envelope compartment in *ΔelyC* cells and as Fe^2+^ crosses the outer membrane passively through porins (49), we hypothesized that chelation of extracytoplasmic iron would be sufficient to suppress the *ΔelyC* PG synthesis arrest and lytic phenotype. To test this, we used ethylenediamine-*N*,*N*′-bis(2-hydroxyphenylacetic) acid (EDDHA), which is known to chelate extracytoplasmic iron (50–53). We used EDDHA and combinations of EDDHA and Fe^2+^ under three different conditions and monitored growth, analyzed PG, measured HO^•^ production, and examined cell morphology at 21°C.

**FIG 5:**
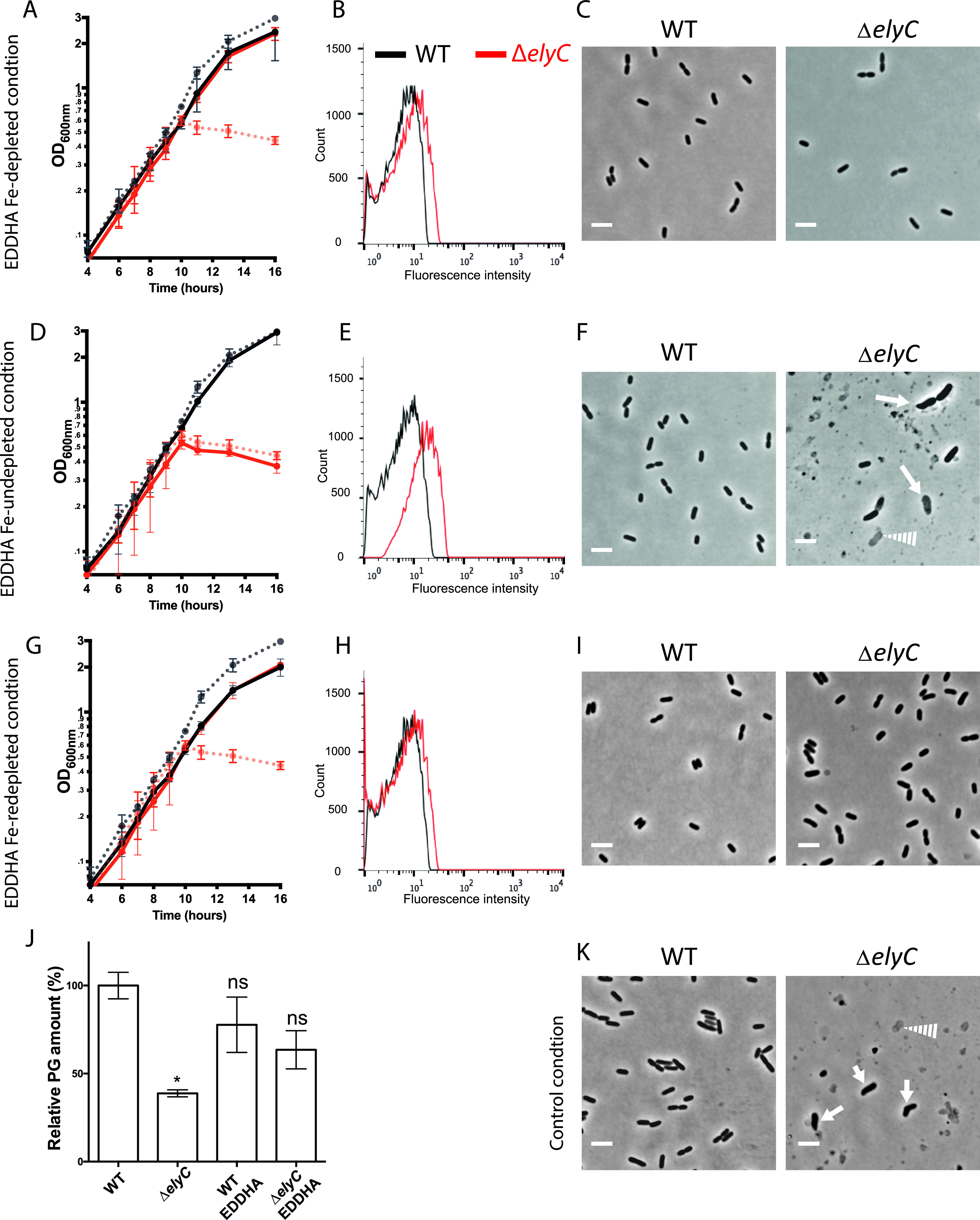
Extracytoplasmic free iron is necessary for Δ*elyC* mutant PG defect and cell lysis at 21°C. (A, D, G) Growth curves of WT (black curves) and Δ*elyC* (red curves) at 21°C. The values represented correspond to the means of OD_600nm_ measurements of at least three biological replicates ± SD. Solid growth curves represent bacteria grown under the condition annotated on the left of the figure. Bacteria grown in control conditions are represented as dotted lines. (B, E, H) Flow cytometry histograms using HPF probe for measuring HO^•^ production in cells grown at 21°C at an OD_600nm_ of 0.35, with WT in black and Δ*elyC* in red. (C, F, I, K) Microscopy photographs of WT and Δ*elyC* cells at ∼14 h of culture. Solid arrows point to lysing or misshaped cells. Dotted arrows point to bacterial ghosts (100× phase contrast objective). Bar = 3 μm. The corresponding growth conditions are indicated on the left: ethylenediamine-*N*,*N*′-bis(2-hydroxyphenylacetic) acid (EDDHA) Fe-depleted condition (culture supplemented with 250 μM EDDHA) (A to C); EDDHA Fe-undepleted condition (culture supplemented with 250 μM EDDHA and 100 μM FeSO_4_) (D–F); EDDHA Fe-redepleted condition (culture supplemented with 100 μM FeSO_4_ and 600 μM EDDHA) (G–I); Relative PG in amount of WT and Δ*elyC* cells grown under control conditions or in EDDHA Fe-depleted condition (EDDHA) (J); and control conditions (K). The values are the means ± SEM (of three biological replicates). The asterisk indicates significant differences to WT grown without EDDHA (*P* < 0.05). ns, not significant.

First, we determined the minimal EDDHA concentration required to suppress Δ*elyC* mutant lysis. The so-called “EDDHA Fe-depleted condition” was obtained by adding EDDHA to the medium at a final concentration of 250 μM. Importantly, under these conditions, the Δ*elyC* mutant grew as well as the WT, without any defect in its growth kinetics (Fig. 5A). As EDDHA does not cross the inner membrane and does not chelate free cytoplasmic iron, it did not cause any growth defect to Δ*elyC* or WT. As expected, in the EDDHA Fe-depleted condition, HO^•^ production and cell morphology were similar between Δ*elyC* and WT cells (Fig. 5B and C and Fig. S2, S3, and S6C). The addition of EDDHA also restored PG synthesis in the Δ*elyC* (Fig. 5J and Fig. S9A to C). These results support our hypothesis that extracytoplasmic iron chelation prevents lysis of the mutant by inhibiting the Fenton reaction occurring in the envelope compartment of Δ*elyC* cells.

To verify these results, we titrated the minimal Fe^2+^ concentration capable of reverting the effect of the extracytoplasmic chelator, thus recreating Δ*elyC* mutant lysis when added to the EDDHA Fe-depleted condition. We found that addition of 100 μM Fe^2+^ to the EDDHA-containing medium (EDDHA Fe-undepleted condition) recreated the mutant lysis phenotype (Fig. 5D). Consistent with this result, the mutant HO^•^ production in EDDHA Fe-undepleted condition increased back to control condition levels (Fig. 5E and Fig. S6C), and lysis was observed under the microscope (Fig. 5F and Fig. S3). Interestingly, compared to the control condition, we also observed an increase in cell length, width, and volume in the mutant but not in the WT (Fig. 5I and Fig. S7A to E).

To further assess the involvement of free extracytoplasmic Fe^2+^ in Δ*elyC* mutant cell lysis, we developed a third condition referred to as the EDDHA Fe-redepleted condition, in which the lytic phenotype of the mutant should be alleviated, as in the first EDDHA Fe-depleted condition. We observed that the Δ*elyC* mutant could indeed be rescued from lysis with 600 μM EDDHA in the presence of 100 μM Fe^2+^. In EDDHA Fe-redepleted conditions, the Δ*elyC* and WT growth kinetics appeared similar, as well as their HO^•^ production (Fig. 5G and H and Fig. S6C). The mutant cells were slightly larger than WT cells, but no other morphological defects could be observed (Fig. 5I and Fig. S2 and S3). Altogether, these results support the idea that extracytoplasmic free iron plays a key role in HO^•^ overproduction, PG biogenesis arrest, and the lytic phenotype of the Δ*elyC* mutant.

## DISCUSSION

Critical to a better understanding of the bacterial cell envelope is a detailed appreciation of the processes that influence its assembly and dynamics. Here, through the characterization of the Δ*elyC* mutant, we reveal that PG synthesis, a major pathway in cell envelope assembly and drug target in the treatment of bacterial infections, can be affected by ROS production. Building our on previous work, our data support a model whereby, in cells lacking ElyC, damaging HO^•^ produced via the Fenton reaction result in protein aggregation in the periplasm, inhibition of PG synthesis, and cell lysis.

Several lines of evidence reported in this study support the idea that ROS are produced mainly in the envelope of *elyC* mutant and not in the cytoplasm, where they lead to toxic accumulation of HO^•^ via de Fenton reaction, inhibition of PG synthesis, and cell lysis. First, we demonstrate that the Δ*elyC* mutant phenotypes are suppressed in anaerobic conditions (Fig. 1). Second, we show that the mutant PG synthesis and lysis defect are suppressed by the iron chelator 2,2′-dipyridyl (Fig. 3) and the extracytoplasmic iron chelator EDDHA (Fig. 5), which inhibit the Fenton reaction. Third, we show that despite the fact that the Δ*elyC* mutant produces significantly higher levels of HO^•^ compared to WT grown in the presence of potassium chromate, it did not activate the SOS response. Thus, while it is formerly possible that smalls amount of HO^•^ are produced in the cytoplasm of the Δ*elyC* mutant, our data are consistent with the idea that most HO^•^ is produced in the envelope of mutant cells, where it quickly reacts and causes damage to the PG synthetic machinery. Although we cannot fully exclude the possibility that HPF probe is activated by peroxynitrite, our data indicate that the overactivation of the HPF probe in the Δ*elyC* mutant is mostly due to HO^•^ overproduction (peroxynitrite is rapidly transformed into nitrogen dioxide in the presence of free Fe^2+^ *in vivo*) (54–56). If the HPF probe were overactivated by peroxynitrite in the Δ*elyC* mutant, adding iron chelator would have increased the probe activation, and this was not the case; the addition of iron chelator to the medium resulted in reduction of HPF activation (Fig. 3).

Previous work indicates that HO^•^ is too reactive to diffuse more than few nanometers (24) and is not likely able to cross a membrane without reacting with a cellular component (57, 58). Thus, HO^•^ can alter only molecules in very close proximity to where the Fenton reaction occurs. In further support of the notion that HO^•^ production is localized in the envelope of the Δ*elyC* mutant, we show that the addition of potassium chromate induces the cytoplasmic SOS response and its characteristic cell filamentation phenotype in Δ*elyC* mutant cells, when the iron chelator 2,2′-dipyridyl is present to annihilate the native production of HO^•^ (Fig. 4 and Fig. S4). This important result indicates that the Δ*elyC* mutant can exhibit two separate ROS-induced phenotypes, based on the location of the source of ROS: (i) an SOS response-independent PG synthesis arrest and cell lysis phenotype that depends on HO^•^ production in the envelope and can be suppressed by the inner membrane impermeable iron chelator EDDHA (Fig. 5) and (ii) an SOS response-dependent, cell filamentation phenotype that depends on ROS production in the cytoplasm and is only apparent in the mutant if the primary phenotypes (PG synthesis inhibition and cell lysis) are suppressed by iron chelation (Fig. 4). We note that while our current study focused on HO^•^ production, it would be interesting to investigate whether other ROS species are also present in the Δ*elyC* mutant and whether they contribute to its phenotypes.

Although the exact function of ElyC remains unclear, it is tempting to speculate on its role and how it contributes to maintaining ROS homeostasis in the envelope at lower temperatures. One hypothesis is that ElyC functions in the stabilization of the electron transport chain (ETC) components at lower temperatures. It has been shown that at lower temperatures, there is increase in saturated lipids, which in turn impacts membrane fluidity (59, 60). Changes in membrane fluidity have been shown to reduce the diffusion of proteins in the ETC (61), which may lead to leakage of electrons from the ETC chain components. This uncontrolled leakage of electrons to molecular oxygen from the ETC in the envelope would subsequently generate superoxide and hydrogen peroxide. Through reactivity with iron sulfur cluster-containing proteins (e.g., cytochromes) in the envelope, this toxic cascade would lead to the liberation of free iron, thus feeding the Fenton reaction and generating HO^•^ in the envelope. The formation of HO^•^ would then lead to protein damage and aggregation, directly or indirectly, inhibiting PG synthesis and therefore causing bacterial cell lysis. Consistent with this overarching hypothesis, (i) we previously showed that the Δ*elyC* mutant PG synthesis arrest and cell lysis phenotype can be suppressed by the overexpression of periplasmic chaperones (but not by cytoplasmic chaperones) (11), and (ii) we now show that anaerobic conditions, as well as the addition of iron chelators, suppress these Δ*elyC* defects (Fig. 3 and 5 and Fig. S6 to S9). Interestingly, suppression of the Δ*elyC* phenotype by overexpression of genes involved in PG synthesis reduced HO^•^ production compared to the Δ*elyC* mutant control (Fig. S1). We note that even in these overexpression strains, the Δ*elyC* mutant background still resulted in higher HO^•^ levels compared to the otherwise-WT cells. That the PG synthesis and cell lysis defects of the Δ*elyC* mutant can be suppressed, despite the higher levels of HO^•^ production, suggests that the PG synthesis and cell lysis phenotypes are an indirect effect of HO^•^ production. Which step in the PG synthetic pathway is susceptible to HO^•^ overproduction remains an outstanding question. Several possible scenarios exist. In the simplest scenario, the PG synthases that act in the periplasm are subject to HO^•^-induced protein aggregation. Alternatively, HO^•^ overproduction results in lipid peroxidation. C55-P, the universal lipid carrier involved in PG synthesis, is a polyunsaturated phospholipid that may be sensitive to ROS levels. Although lipid peroxidation remains poorly studied in bacteria, it is widely described in Eukaryotes as a consequence of ROS (62, 63). Examining lipid peroxidation in the Δ*elyC* mutant may test this hypothesis.

The exact function of ElyC remains unclear. One idea is that ElyC function is likely linked to maintaining ROS homeostasis in the envelope, possibly through stabilization of the ETC complexes, as hypothesized above. Alternative hypotheses in which ElyC functions directly in iron homeostasis in the envelope are also possible. In future work, deciphering the protein-protein interaction network of ElyC may reveal its exact role in envelope biology. Furthermore, transcriptomic studies examining the activity of transcription factors related to oxidative stress (e.g., OxyR, SoxR, NsrR) (64–67) or envelope stress (e.g., CpxR, RpoE, RscA) (68–70) may reveal the extent to which the Δ*elyC* mutant is responding to oxidative damage and envelope stress. Finally, experiments examining the involvement of cytosolic and periplasmic superoxide dismutases or other ROS defenses (e.g., catalases, alkyl hydroperoxide reductases, thiol peroxidase, peroxiredoxin) in the viability of the Δ*elyC* mutant may also reveal whether other ROS are accumulating in the mutant.

Overall, our results provide evidence of a molecular cascade whereby ROS production in the envelope leads to inhibition of PG synthesis and cell lysis. Understanding the mechanistic basis for ROS production in the Δ*elyC* mutant will likely reveal molecular targets in otherwise-WT cells that can lead to the development of new antibiotics, thus contributing to more effective strategies dealing with antibiotic resistant infections in Gram-negative pathogens. Finally, considering that the immune system utilizes ROS as a means to cope with bacterial pathogens, a better understanding of how ROS are generated in the envelope and interfere with PG synthesis could lead to strategies that potentiate the immune ROS effect in the killing of bacteria.

## MATERIALS AND METHODS

### Bacterial stains.

In our study, we compared the phenotypic differences between E. coli K-12 MG1655 WT (71), *ΔelyC* mutant EM9 strain (MG1655, *elyC*::*FRT*) (11), and Δ*mrcB* MM39 strain (MG1655, *mrcB*::*FRT*) (11). For quantification of SOS response activation, we constructed WT Φ(*sulAp*-*lacZ*) corresponding to strain SG24 (MG1655, Δ(*lacI*-*lacZp*)Φ(Kan^R^*-sulAp*-*lacZ*)) and Δ*elyC* Φ(*sulAp*-*lacZ*) corresponding to strain SG25 (EM9, Δ(*lacI*-*lacZp*)Φ(Kan^R^*-sulAp*-*lacZ*)) (this study). In SG24 and SG25, the *lacZ* promoter was replaced by the *sulA* promoter fused with the functional *lacZ* gene, the *lacI* gene was deleted, and the kanamycin resistance gene was added. More information is available in supplemental Materials and Methods.

### Growth conditions and monitoring.

For all experiments involving growing bacteria in liquid medium, the cells were initially grown as overnight cultures in Luria-Bertani (LB) medium at 37°C in culture tubes under agitation on a culture rotator. LB medium was prepared as previously described (18) with 1% peptone (Biobasic), 0.5% yeast extract (Biobasic), and 0.5% NaCl (Sigma-Aldrich), in distilled water. Then, bacteria optical density was measured at 600 nm (OD_600nm_), and day cultures were started from dilution of overnight cultures to OD_600nm_ of 0.025 in LB medium 1% NaCl (Miller’s LB Broth from Biobasic). Under control conditions, bacteria were grown in 25 mL LB 1% NaCl in 250-mL flasks with shaking at 250 rpm (New Brunswick, Innova 4230) at 21°C. For the culture narrow tube condition, bacteria were grown in 5 mL LB 1% NaCl in 10-mL culture tubes under agitation on a culture rotator. Anaerobic growth conditions were achieved by growing bacteria in hermetically closed glass bottles completely filled in with LB 1% NaCl, with the atmospheric air in the medium chased by O_2_-free argon (72).

For Fe-depleted conditions, LB medium was supplemented with 375 μM 2,2′-dipyridyl from Sigma-Aldrich. For EDDHA Fe-depleted conditions, LB medium was supplemented with 250 μM ethylenediamine-*N*,*N*′-bis(2-hydroxyphenylacetic) acid from Alfa Chemistry (catalog number ACM117002). For Fe-undepleted conditions, LB medium was supplemented with 375 μM 2,2′-dipyridyl and 100 μM FeSO_4_ purchased from Sigma. For EDDHA Fe-undepleted conditions, LB medium was supplemented with 250 μM EDDHA and 100 μM FeSO_4_. For Fe-redepleted conditions, LB medium was supplemented with 100 μM FeSO_4_ and 600 μM 2,2′-dipyridyl. For EDDHA Fe-redepleted conditions, LB medium was supplemented with 100 μM FeSO_4_ and 600 μM EDDHA. For potassium chromate treatments, bacteria were grown in the control condition in medium supplemented with 120 μM potassium chromate (K_2_CrO_4_, Sigma-Aldrich).

Absorbance was measured on each sample at 600 nm with a Fisherbrand cell density meter at the indicated time points. Growth curves are representative of at least three biological replicates.

### PG purification and analysis.

PG sacculi were prepared from MG1655 and Δ*elyC* day cultures (see Growth conditions and monitoring) in control, Fe-depleted, and EDDHA Fe-depleted conditions at 21°C. When OD_600nm_ reached 0.5, cultures were centrifuged during 10 min at 4,000 × *g* at 4°C and resuspended in 5 mL phosphate-buffered saline (PBS) buffer. The cells were added, drop by drop, in 10 mL of boiling 5% sodium dodecyl sulfate (SDS, Sigma). Liebig condenser was used to avoid evaporation during boiling steps. The cells were boiled for 2 h, and preparations were cooled overnight at room temperature (73, 74).

PG samples were processed and analyzed as described previously (73, 74). PG sacculi were pelleted by ultracentrifugation for 15 min at 60,000 rpm (TLA100 Beckman rotor, Optima Max-TL Ultracentrifuge, Beckman) and washed three times by repeated cycles of centrifugation and resuspension in Milli-Q water. Afterwards, the samples were digested with pronase E (100 μg/mL) in 10 mM Tris-HCl, pH 7.5 buffer for 1 h at 60°C to remove Braun’s lipoproteins. After addition of SDS 1% (wt/vol) (final concentration), reactions were heat-inactivated, and SDS detergent was removed by further washing steps. The samples were treated with muramidase (100 μg/mL) for 16 h at 37°C, in 50 mM phosphate buffer, pH 4.9. Muramidase digestion was stopped by boiling, coagulated proteins were removed by centrifugation (10 min, 14,000 rpm), and the supernatants were reduced with 15 μL 0.5 M sodium borate, pH 9.5, and sodium borohydride (10 mg/mL final concentration, 30 min at room temperature). Finally, samples (100 μL) were adjusted to pH 3.5 with phosphoric acid.

Ultraperformance liquid chromatography (UPLC) analyses of muropeptides were performed on a Waters UPLC system (Waters Corporation, USA) equipped with an Acquity UPLC BEH C18 column, 130 Å, 1.7 μm, 2.1 mm × 150 mm (Waters, USA). Elution of muropeptides was detected at 204 nm. Muropeptides were separated at 35°C using a linear gradient from buffer A (50 mM phosphate buffer, pH 4.35) to buffer B (50 mM phosphate buffer, pH 4.95, 15% methanol [vol/vol]) in a 20-min run, with a 0.25 mL/min flow.

Identification of each muropeptide was performed by comparison of the retention-times and mass spectrometric data to known samples (73). Quantification was done by integrating peak areas of each muropeptide in the chromatogram and normalized to their molar ratio. Relative total PG amount was calculated by comparison of the total intensities (total area) of the chromatograms from three biological replicates normalized to the same OD_600_/mL. Main PG features were calculated as follows: percentage of monomers, dimers, trimers, and tetramers was calculated by adding the relative molar abundances of the different oligomers; overall cross-link was calculated as dimers + (trimers × 2) + (tetramers × 3); the percentage of anhydro muropeptides was calculated by adding the relative molar abundances of the different anhydro species; and average glycan chain length was calculated by dividing 100 by the percentage of anhydro muropeptides. The samples were analyzed in triplicate, and *t* tests were performed for statistical comparisons.

### Flow cytometry.

Once a culture OD_600nm_ reached 0.35, 1 mL of cells were centrifuged at 12,000 rpm at the respective culture temperature. The cells were washed twice, resuspended in 1 mL of PBS buffer, and transferred to a polycarbonate flow cytometry tube. Then, 2 μl of 2-[6-(4′-hydroxy)phenoxy-3*H*-xanthen-3-on-9-yl]benzoic acid (HPF) (Sigma, number H4290) was added. After 30 min of incubation with HPF, the cells were analyzed in a Becton Dickinson FACScalibur. The threshold was set on forward and side scatter on WT populations grown in control conditions at 21 or 37°C. HPF fluorescence signal settings (excitation/emission: 490/515 nm) threshold were normalized to WT populations grown in control conditions without the HPF probe at 21 or 37°C. At least 30,000 events were acquired per sample.

### β-Galactosidase assay.

SOS response activation through *sulA* promoter fusion with *lacZ* reporter were measured by Miller assay (75). Blocking solution were made of 1 M Na_2_CO_3_, 4 mg/mL *O*-nitrophenyl-β-d-galactopyranoside (ONPG), and 0.1 M sodium phosphate buffer, pH 7. Lysis solution is composed of 25 mL β-mercaptoethanol, 5 mL toluene, 5 mL 0.02 M MnSO_4_, and 5 mL 10% SDS. The blank consisted of 500 μl 1 M Na_2_CO_3_, 800 μl sodium phosphate buffer, 200 μl 4 mg/mL ONPG, and 50 μl lysis solution. The cells were grown until OD_600nm_ of 0.35 and cooled on ice for 10 min. The cells were lysed using lysis buffer for 10 min at 28°C and incubated at 28°C with ONPG solution and sodium phosphate buffer until coloration appeared. The reaction was stopped using 500 μl of the Na_2_CO_3_ solution. The enzyme activities were represented in Miller units (75), and OD_420nm_ and OD_550nm_ were measured with Beckman Coulter DU-64 UV-visible spectrophotometer.

### Microscopy.

For bacterial morphology observations, 1 μl of fresh culture was applied to an agarose pad on a slide and covered by a coverslip. Images were captured on a Nikon Eclipse 80i with Plan Fluor 100× oil Ph3 DLL objective and a Nikon DS-Ri2 color camera.

### CPRG assay.

The CPRG phenotype was observed on CPRG agar plates as described previously (11, 18). One colony of each strain was plated to form an X and incubated overnight at 21°C under aerobic or anaerobic conditions. The CPRG agar plates were made of LB broth, 1.5% agar (Biobasic), 20 mg/mL chlorophenol red-b-D-galactopyranoside (CPRG), and 50 μM isopropyl-β-d-thiogalactopyranoside (IPTG). Anaerobic conditions for CPRG agar plates were created with a BD GazPak system.

### Statistical analysis.

GraphPad PRISM Software, Inc. (San Diego, CA, www.graphpad.com) was used for all statistical analyses. To determine significance of the data, *t* test (unpaired) or analysis of variance (ANOVA) was performed. All experiments were performed at least in biological triplicates.
