# Supplementary material for: Hydroxyl Radical Overproduction in the Envelope: an Achilles’ Heel in Peptidoglycan Synthesis
Source: Microbiol Spectr. 2022 Feb 16;10(1):e01203-21. doi: 10.1128/spectrum.01203-21 (PMC8849086; doi:10.1128/spectrum.01203-21)
Supplement: SUPPLEMENTAL FILE 1 — Supplemental material. Download SPECTRUM01203-21_Supp_1_seq5.pdf, PDF file, 1.4 MB [file spectrum01203-21_supp_1_seq5.pdf]

Supplementary Information for

Hydroxyl radical overproduction in the envelope: an Achilles' heel in peptidoglycan synthesis

Sean Giacomucci[*†], Laura Alvarez[‡], Christopher D. A. Rodrigues[§], Felipe Cava[‡], and Catherine Paradis-Bleau[*†].

(**†**): Catherine Paradis-Bleau Laboratory, Département de microbiologie, infectiologie et immunologie, faculté de médecine, locaux R-615/616, Université de Montréal C.P. 6128, Succ. Centre-ville Montréal (Québec) H3C 3J7 ; sean.giacomucci@umonrteal.ca, catherine.paradis-bleau@umontreal.ca.

(**‡**): Felipe Cava Laboratory, Molecular Infection Medicine Sweden (MIMS) and Umea Center for Microbial Research (UCMR), Umea University, Laboratory 6k-146. Office 6L-142, SE-901 87 Umeå. Sweden; laura.alvarez@umu.se; felipe.cava@ umu.se.

(**§**): Christopher Rodrigues Laboratory, The ithree Institute, University of Technology Sydney (UTS), Sydney NSW, Australia, christopher.rodrigues@uts.edu.au.

This PDF file includes:

Supplementary Materials and Methods, including Table S1
Figures S1 to S9 and Table S2
SI References

Supplemental Materials and Methods

Batch measurement of cell aera and width using ImageJ.

For the different conditions, at least 100 cells were measured. Cells were counted using ImageJ (v. 1.53i). Images were converted and treated with the following macro in ImageJ Batch Process utility.

```
setOption("ScaleConversions", true);
run("16-bit");
setAutoThreshold("Default");
//run("Threshold...");
	setAutoThreshold("Default");
	run("Convert to Mask");
```

Then, cells measurment were batch processed using the following macro in ImageJ Batch Process utility.

```
title = getTitle;
path = getInfo("image.directory");
print(title);
print(path);
//ids[i]=getImageID;
run("Analyze Particles...", "size=1-10 circularity=0.00-.9 display excludes clear summarize");
///For bacteria grown in presence of chromate
///run("Analyze Particles...", "size=1-50 circularity=0.00-.6 display excludes clear summarize");
selectWindow("Results");
saveAs("Results", path+title+".csv");
close("Results");
close("Summary");
close("log");
```

Statistical analysis were processed using Graphpad® Prism 8.

Strains and Vectors Construction.

Plasmid pSG3 (

Table **S1**) was constructed from the pKD13 (1) derivative; vector pCB126 (

Table **S1**). The *sulA* promoter was amplified on *E. coli* MG1655 with primers “PsulA-SalI-FWD” and “PsulA-EcoRI-REV”. Then, pCB126 and the SalI-*sulA*-EcoRI PCR fragment (

Table **S1**) were digested with the SalI and EcoRI enzymes. Ligation was performed with T4 ligase. *E. coli* DH5α was transformed with pSG3 using the Chung protocol (2). The recombining fragment "Δ*lacI-lacZp_kanR_sulAp-lacZ* " containing: -107 to -68 bp upstream of the native *lacI* ORF region, kanamycin resistance gene, *sulA* promoter and -16 to +24 bp of the *lacZ* ORF, was constructed by overlap extension with primers "FusionPromLac-For" and "FusionPromLac-Rev" using pSG3 as template. SG23 strain was constructed by electroporation of recombinant fragment "Δ*lacI-lacZp_kanR_sulAp-lacZ* " on *λ* red recombinase competent *E. coli* TB10 strain (3), with D. Yu *et al.* protocol (4). MG1655 and EM9 strains were transduced with SG23 P1 phage lysate were, creating respectively SG24 and SG25 strains. SG23, SG24 and SG25 strains containing the *sulA* promoter fusion with *lacZ* were verified by PCR and by Sanger sequencing using primer "*kanR*-forward" and "*lacZ*-reverse". Restriction enzymes and T4 ligase were purchased from New England Biolab® Ltd, mini-prep kit were purchased from Fermentas®, kanamycin from Biobasic©, and JumpStart® taq polymerase from Sigma-Aldrich®.

Table S1. Table of strains, vectors and amplification fragments

Strain	Genotype	Source
MG1655	*rph1 ilvG rfb-50*	(5)
"Δ*elyC"*, EM9	MG1655, *elyC*::FRT	*(6)*
"Δ*mrcB"*, MM39	MG1655, *mrcB*::FRT	*(6)*
TB10	MG1655*, nadA::*Tn10 *λcI857 Δ*(*cro-bioA*)	(3)
SG23	TB10, Δ(*lacI-lacZp*)Φ(Kan^{R}-*sulAp-lacZ*)	*This study*
SG24	MG1655, Δ(*lacI-lacZp*)Φ(Kan^{R}-*sulAp-lacZ*)	*This study*
SG25	EM9, Δ(*lacI-lacZp*)Φ(Kan^{R}-*sulAp-lacZ*)	*This study*

Vectors	Features	Origin	Source
pKD13	Amp^{R}, rgnB(Ter), tL3λ(Ter)	R6Kγ	(1)
pCB126	Amp^{R}, *rgnB*(Ter), *sodA*P, tL3λ(Ter)	R6Kγ	(6)
pSG3	Amp^{R}, *rgnB*(Ter), *sulA*P, tL3λ(Ter)	R6Kγ	*This study*
pMob-uppS	Amp^{R}, *lacI^{q}*, *tacP*::*uppS*	pBR322	(6)
pMob-sanA	Amp^{R}, *lacI^{q}*, *tacP*::*sanA*	pBR322	(6)

pMob-murA	Amp^R, *$lacI^q$*, *tacP*::*murA*	pBR322	(6)

PCR fragment	Template	Primers	Source
SalI-*sulAP*-EcoRI	*E. coli* MG1655	Psfia-SalI-FWD<br>PsfiA-EcoRI-Rev	*This study*

Primers.

Oligonucleotides were purchased from BioCorp®.

PsulA-SalI-FWD: ATCGATGTCGACCGAGGCTCTTTCCGAAAATAGGGT

PsulA-EcoRI-REV: GCGCATGAATTCAATCAATCCAGCCCCTGTGAGTTAC

FusionPromLac-For: CGGAAGGCGAAGCGGCATGCATTTACGTTGACACCATCGTTGAGCGATTGTGTAGGCTG

FusionPromLac-Rev: CAGTGAATCCGTAATCATGGTCATAGCTGTTTCCTGTGTGTATCGTGAGGATGCGTCATC

kanR-forward: ATTCATCGACTGTGGCCGGC

lacZ-reverse: AGTTGGGTAACGCCAGGGTT

Supplementary Figures and table

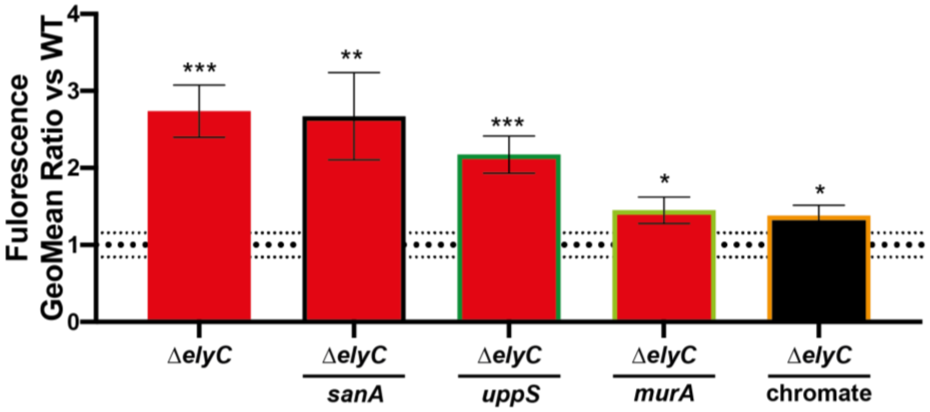

Figure S1. Effect of *uppS* and *murA* overexpression on HO$^{\bullet}$ production in Δ*elyC* mutant.

Overexpression of the *uppS* and *murA* genes partially reduces HO$^{\bullet}$ production in Δ*elyC* mutant cells. Relative HPF fluorescence intensity geomean of WT and Δ*elyC* cells, relative to the WT control grown at 21°C. All strains were cultivated in control condition (see materials and methods and supplemental material and methods), cells carrying a vector were grown in presence of 100 µM IPTG. Overexpression of the *sanA* gene coding for another DUF218-containing protein in *E. coli* represents a negative control in the assay as it was shown to have no impact on Δ*elyC* mutant phenotype (6). All experiments were performed in at least 3 biological replicates except for overexpression of *murA* which was performed in duplicate. Large dotted lines represent WT control fluorescence geomean ratio and the small dotted line represents its confidence interval. Culture conditions and vector construction are indicated in materials and methods and supplemental material and methods. Error bar represents geomean confidence intervals. Ordinary one-way ANOVA with Graphpad© Prism 8, ns(P>0.05), *(P>0.05), **(P>0.01), ***(P>0.001) and ****(P=0.0001).

89

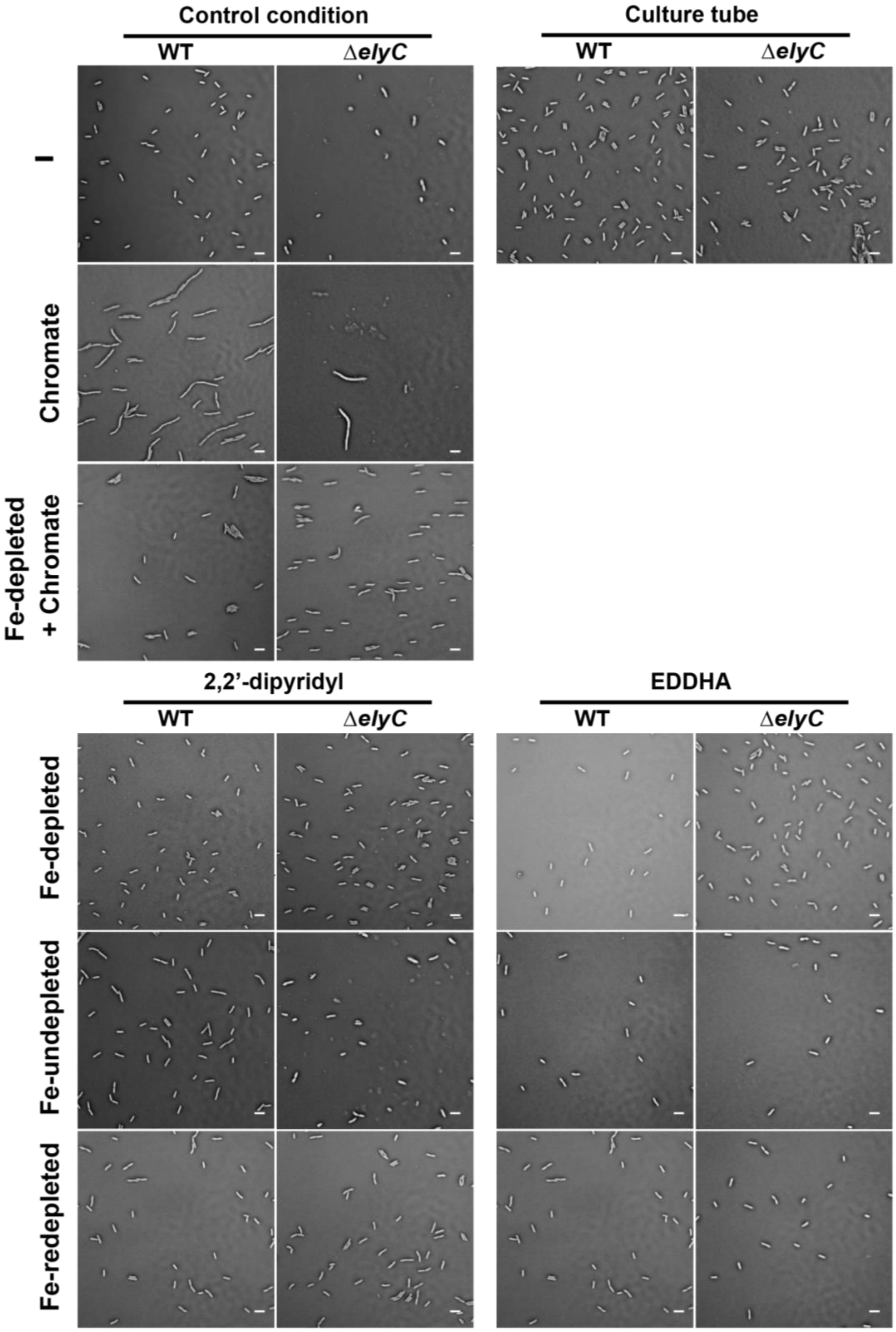

Control condition
WT
ΔelyC
Culture tube
WT
ΔelyC
-
Chromate
Fe-depleted + Chromate
2,2'-dipyridyl
WT
ΔelyC
EDDHA
WT
ΔelyC
Fe-depleted
Fe-undepleted
Fe-redepleted

90

Figure S2. Representative microscopy images, showing larger panels of cells grown in different conditions.

Microscopy photographs of fresh WT and Δ*elyC* mutant cells when OD_{600} reached 0.35 in the different conditions except for Δ*elyC* cells grown with chromate (OD_{600nm} ~ 0.20 during lysis at ~8 hours, when WT cells grown with chromate reached OD_{600nm} of 0.35). Fe-depleted condition (cultures supplemented with 375 µM 2,2'-dipyridyl)**,** Fe-undepleted condition (cultures supplemented with 375 µM 2,2'-dipyridyl and 100 µM $FeSO_4$)**,** Fe-redepleted condition (cultures supplemented with 600 µM 2,2'-dipyridyl and 100 µM $FeSO_4$)**,** chromate condition (cultures supplemented with 125 µM potassium chromate), Fe-depleted + chromate condition (cultures supplemented with 375 µM 2,2'-dipyridyl and 125 µM potassium chromate)**,** EDDHA Fe-depleted condition (culture supplemented with 250 µM EDDHA), EDDHA Fe-undepleted condition (culture supplemented with 250 µM EDDHA and 100 µM $FeSO_4$); EDDHA Fe-redepleted condition (culture supplemented

with 100 µM $FeSO_4$ and 600 µM EDDHA)**.** Culture conditions are indicated in materials and methods. Scale bars = 5 µm.

107

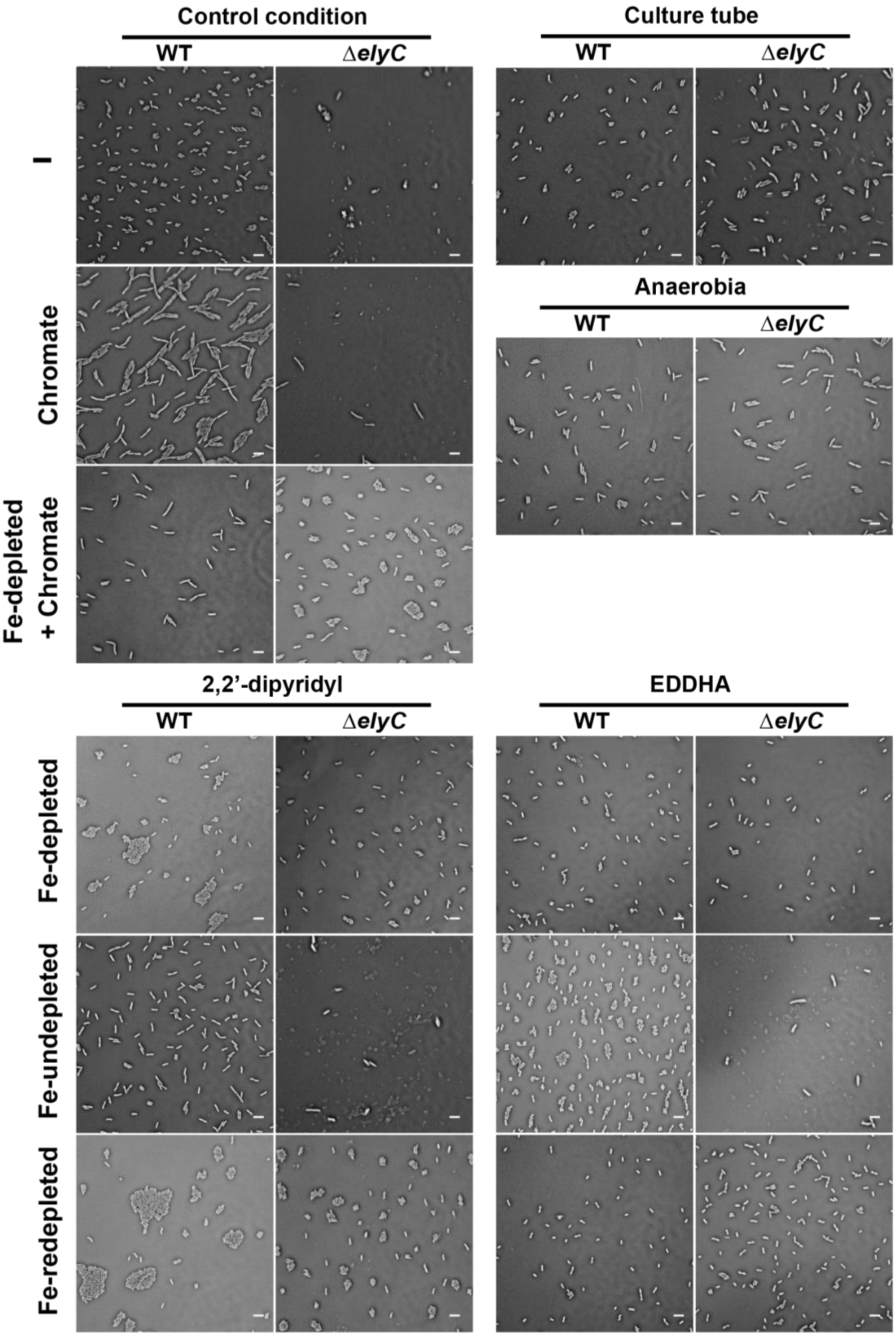

Control condition
WT
ΔelyC
Culture tube
WT
ΔelyC
I
Chromate
Anaerobia
WT
ΔelyC
Fe-depleted + Chromate
2,2'-dipyridyl
WT
ΔelyC
EDDHA
WT
ΔelyC
Fe-depleted
Fe-undepleted
Fe-redepleted

Figure S3. Representative microscopy images, showing larger panels of cells grown in different conditions for an extended period of time.

Microscopy photographs of fresh WT and ΔelyC mutant cells cultures after 14 hours in the different conditions, except for anaerobia (after 24 hours, OD600nm ~0.5). Fe-depleted condition (cultures supplemented with 375 µM 2,2'-dipyridyl); Fe-undepleted condition (cultures supplemented with 375 µM 2,2'-dipyridyl and 100 µM FeSO4); Fe-redepleted condition (cultures supplemented with 600 µM 2,2'-dipyridyl and 100 µM FeSO4); chromate condition (cultures supplemented with 125 µM potassium chromate); Fe-depleted + chromate condition (cultures supplemented with 375 µM 2,2'-dipyridyl and 125 µM potassium chromate); EDDHA Fe-depleted condition (culture supplemented with 250 µM EDDHA); EDDHA Fe-undepleted condition (culture supplemented with 250 µM EDDHA and 100 µM FeSO4); EDDHA Fe-redepleted condition (culture supplemented with 100 µM FeSO4 and 600 µM EDDHA). Culture conditions details are indicated in materials and methods. Scale bars = 5 µm.

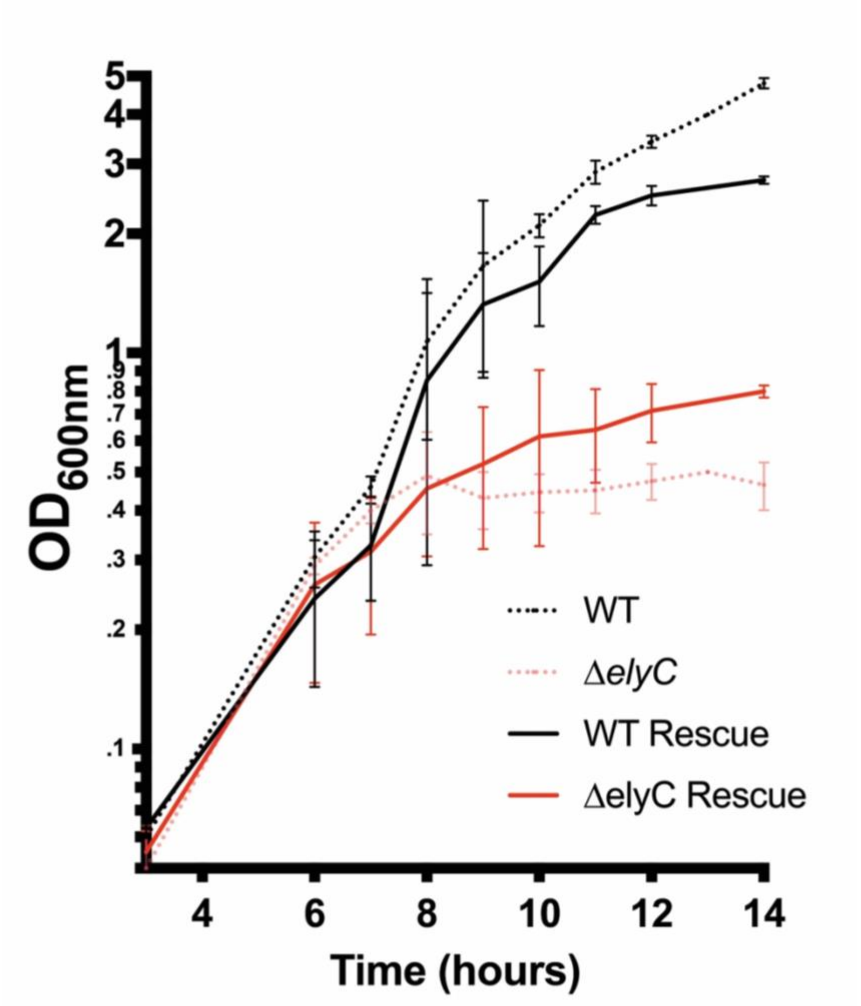

Figure S4. Δ*elyC* cell lysis requires high levels of $HO^{\bullet}$.

Growth curves of WT (black curves) and Δ*elyC* (red curves) cells grown in control condition (dotted fainted lines) and with the addition of 375 µM 2,2'-dipyridyl after 6 hours (solid lines, "Rescue"). Values represented correspond to the mean of OD_{600nm} measurements on, at least, 3 biological replicates ± SD. Adding 2,2'-dipyridyl 6 hours after growth initiation rescued the mutant from lysis. Culture conditions are indicated in materials and methods.

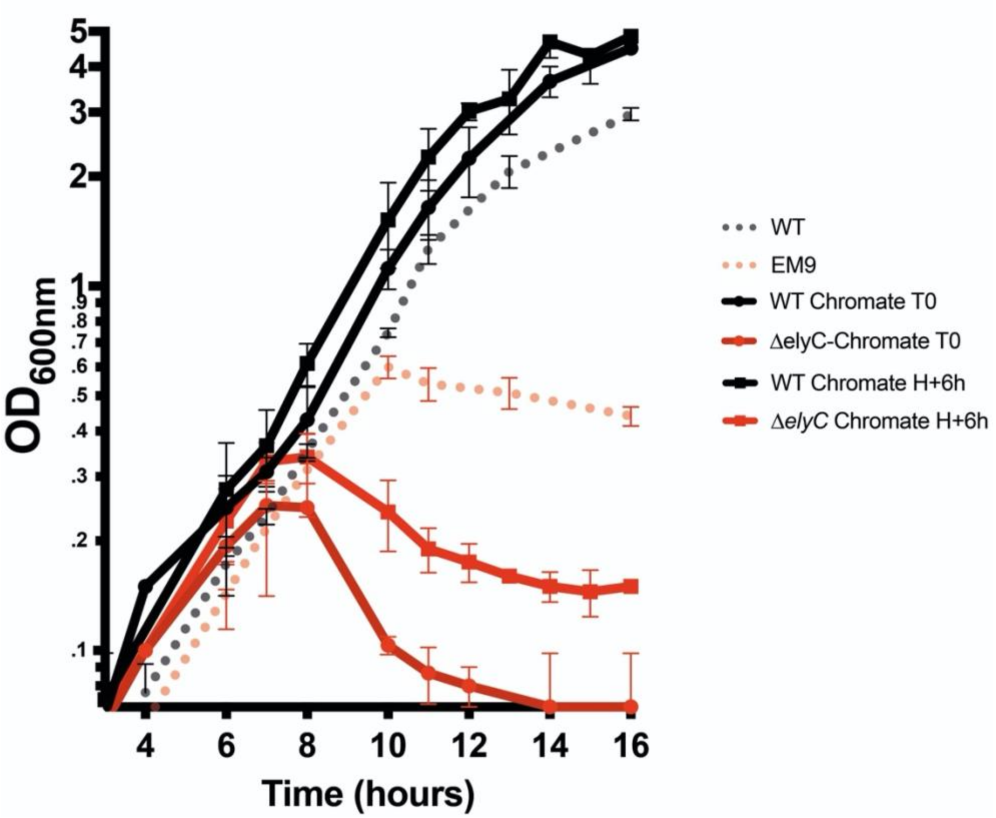

Figure S5. Exogenous HO$^{\bullet}$-generating potassium chromate induces a rapid and exacerbated lysis in Δ*elyC* cells.

Growth curves of WT (black curves) and Δ*elyC* (red curves) cells grown in control condition (dotted fainted lines) or grown in presence of 125 µM potassium chromate (solid curves), values represented correspond to the mean of OD_{600nm} measurements on, at least, 3 biological replicates ± SD. Chromate were added at growth initiation (T0) or was added 6 hours after growth initiation (H+6h). Adding potassium chromate to Δ*elyC* mutant cells at both growth initiation or 6 hours after growth initiation accelerated and worsened the lysis phenotype. Culture conditions are indicated in materials and methods. Representative growth curves of biological a triplicate.

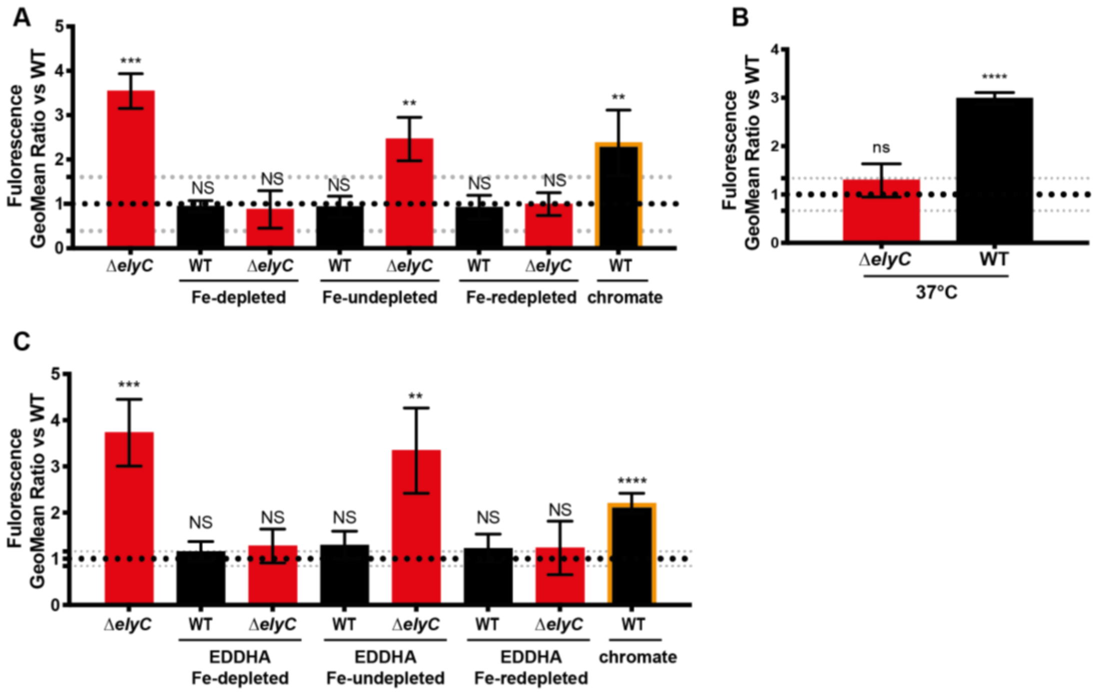

Figure S6. Statistical analysis of WT and Δ*elyC* cells HPF fluorescence intensity. (A-C) Relative HPF fluorescence intensity geomean of WT and Δ*elyC* cells, relative to the corresponding WT control (control condition at 21°C or 37°C). Large dotted lines represent WT control fluorescence geomean ratio and the small dotted line its confidence interval. Error bar represents confidence intervals. (A) Bacteria grown at 21°C in control condition, Fe-depleted condition (cultures supplemented with 375 µM 2,2'-dipyridyl)**;** Fe-undepleted condition (cultures supplemented with 375 µM 2,2'-dipyridyl and 100 µM $FeSO_4$)**;** Fe-redepleted condition (cultures supplemented with 600 µM 2,2'-dipyridyl and 100 µM $FeSO_4$); chromate condition (cultures supplemented with 125 µM potassium chromate) as indicated in material and methods. Cells grown in presence of chromate were used as $HO^{\bullet}$ overproducing cells positive control in the cytometry assay. (B) Bacteria grown at 37°C in control condition or with chromate for positive control. (C) Bacteria grown at 21°C in control condition, with chromate for positive control; EDDHA Fe-depleted condition (culture supplemented with 250 µM EDDHA); EDDHA Fe-undepleted condition (culture supplemented with 250 µM EDDHA and 100 µM $FeSO_4$); EDDHA Fe-redepleted condition (culture supplemented with 100 µM $FeSO_4$ and 600 µM EDDHA) as indicated in material and methods. Ordinary one-way ANOVA were performed with Graphpad© Prism 8, ns(*P*>0.05), *(*P*>0.05), **(*P*>0.01), ***(*P*>0.001) and ****(*P*=0.0001).

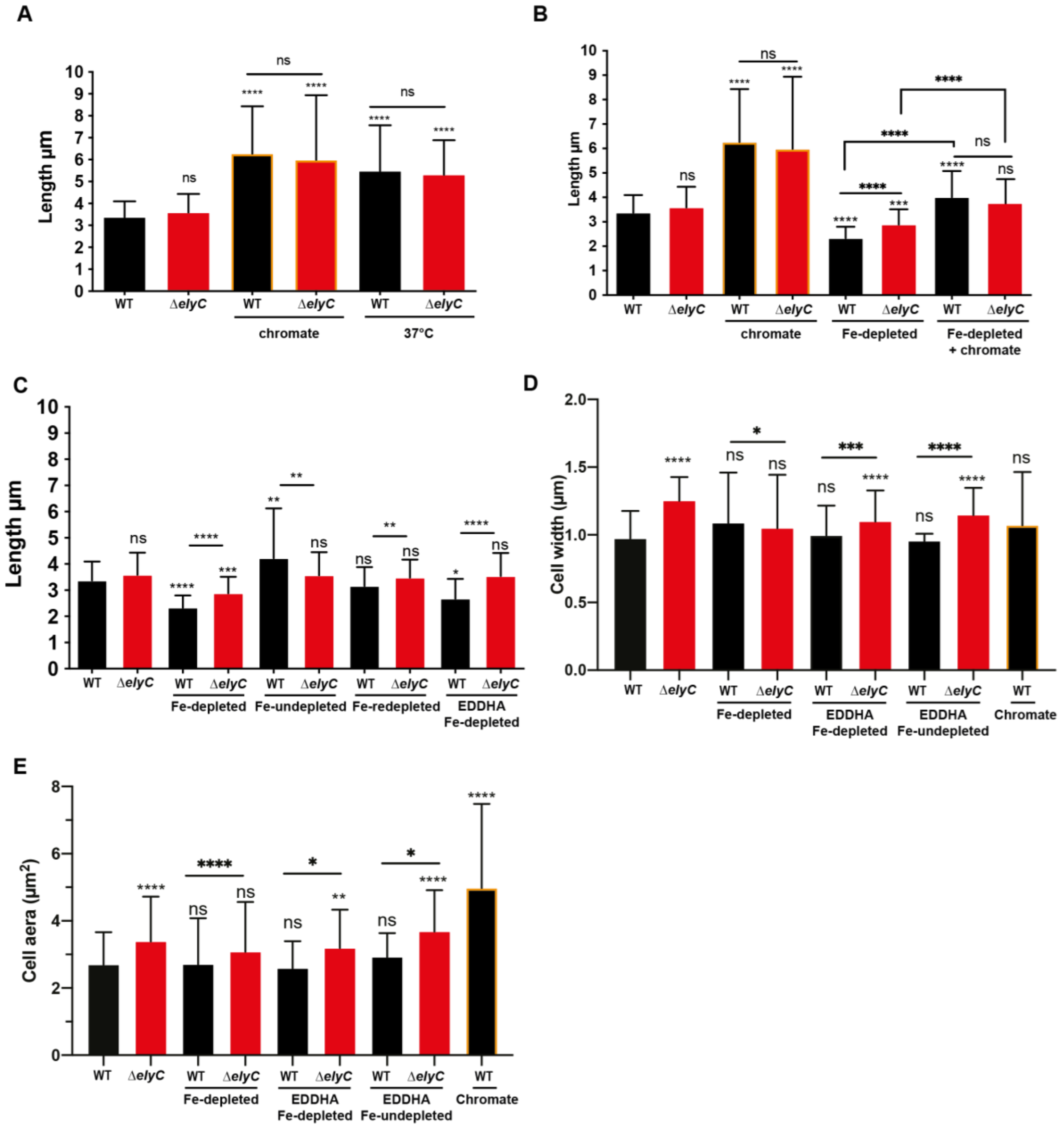

Figure S7. Bacterial cell length, width and surface area analysis.

Means ± SD of WT and Δ*elyC* cell length, width and surface in different conditions. No specification indicates that strains were grown in control conditions at 21°C. Measurements were performed on microscopy images collected at an OD_{600nm} of 0.3 using ImageJ, see material and methods for culture conditions and supplemental material and methods for microscopy image analysis protocol. Cell length, width or surface area were compared to the WT grown in control condition. In this case statistical significance is represented by

stars or “NS” directly above the corresponding error bar. For comparisons between WT and mutant cells in the same condition, the statistical significance is indicated on top of the black line, placed above the corresponding bars. For facilitating values comparisons, measurements on WT and Δ*elyC* cells grown in control condition were systematically reported on each graph. (A–C) Mean cell length (µm ± SEM) and cells were collected at OD_{600nm} of 0.35. (A) Mean cell length in control condition at 21°C, in presence of 125 µM potassium chromate (chromate) or grown at 37°C. Measurement of Δ*elyC* mutant cells grown with chromate does not include lysed cells and cells were collected when WT cells reached $OD_{600nm} = 0.3$. (B) Effects of chromate and/or 375 µM 2,2’-dipyridyl (Fe-depleted) on cell length. (C) Mean cell length in Fe-depleted condition**,** Fe-undepleted condition (cultures supplemented with 375 µM 2,2’-dipyridyl and 100 µM $FeSO_4$)**,** Fe-redepleted condition (cultures supplemented with 600 µM 2,2’-dipyridyl and 100 µM $FeSO_4$) and EDDHA Fe-depleted condition (culture supplemented with 250 µM EDDHA). (D) Mean cell width (µm ± SEM) in control condition; Fe-depleted condition; chromate condition; EDDHA Fe-depleted condition; EDDHA Fe-undepleted condition. (E) Mean cell area ($µm^2$ ± SEM) of WT and Δ*elyC* strain in control condition, Fe-depleted condition, chromate condition, EDDHA Fe-depleted condition and EDDHA Fe-undepleted condition. Ordinary one-way ANOVA were performed with Graphpad© Prism 8, ns($P>0.05$), *($P>0.05$), **($P>0.01$), ***($P>0.001$) and ****($P=0.0001$).

A

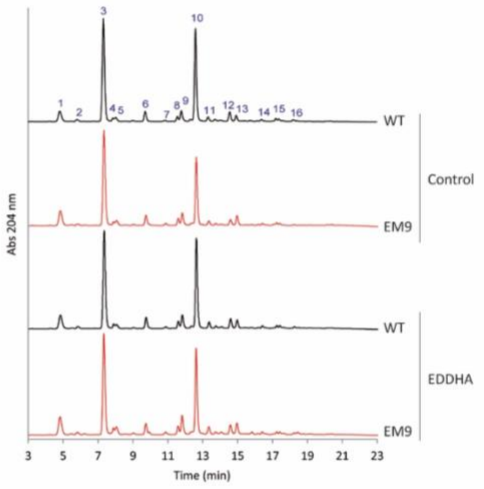

B

Parameter	wt		EM9		wt EDDHA		EM9 EDDHA	
	%	SD	%	SD	%	SD	%	SD
Monomers	69.5	0.3	73.7 *	0.6	68.7	0.5	69.9	3.3
Dimers	29.5	0.3	24.7 *	0.6	29.9	0.5	28.5	2.7
Trimers	1.0	0.0	1.6 *	0.0	1.3	0.1	1.6	0.6
Di	1.0	0.1	1.1	0.1	1.5 *	0.1	2.0	0.9
Tri	12.6	0.5	16.8 *	0.4	15.2 *	0.5	16.7	1.6
Tetra	83.4	0.5	78.4 *	0.3	80.2 *	0.4	75.8 *	1.5
Penta	3.0	0.1	3.7	0.3	3.1	0.1	5.4	2.1
Lpp	7.1	0.3	6.6	0.3	7.5	0.2	6.6	0.3
Anhydro	1.4	0.2	1.0	0.3	1.6	0.1	1.3	0.2
Crosslink	31.5	0.4	27.9 *	0.7	32.6	0.6	31.6	3.8

C

Peak	Muropep.	wt		EM9		wt EDDHA		EM9 EDDHA	
		%	SD	%	SD	%	SD	%	SD
1	M3	7.5	0.5	11.1 *	0.3	9.5 *	0.4	11.4 *	1.4
2	$M4^G$	1.1	0.2	0.7	0.1	0.8	0.5	1.2	0.4
3	M4	53.1	0.3	52.9	1.0	49.9 *	0.3	46.7 *	1.2
4	M2	1.0	0.1	1.1	0.1	1.5 *	0.1	2.0	0.9
5	M5	2.1	0.1	2.7	0.3	1.8	0.1	3.9	2.2
6	M3-Lpp	4.8	0.2	5.2	0.4	5.4	0.2	4.7	0.3
7	D33	0.2	0.0	0.5 *	0.0	0.4 *	0.0	0.6 *	0.1
8	D34	1.0	0.1	1.5 *	0.1	1.4 *	0.1	1.6	0.4
9	D43	2.7	0.0	3.1	0.2	3.2	0.1	4.1	0.9
10	D44	21.1	0.3	16.3 *	0.2	20.0	0.4	17.5 *	1.3
11	D45	0.9	0.1	1.0	0.0	1.3	0.2	1.5 *	0.1
12	D43-Lpp	2.2	0.0	1.4 *	0.1	2.1	0.1	1.9 *	0.0
13	T444	0.9	0.0	1.5 *	0.1	1.1 *	0.0	1.4	0.5
14	D43N	0.3	0.1	0.4	0.1	0.5	0.0	0.4	0.1
15	D44N	0.9	0.1	0.6	0.2	1.0	0.2	0.7	0.0
16	T444N	0.2	0.0	0.1	0.0	0.2	0.1	0.2	0.1

Figure S8. Wild type and Δ*elyC* cells peptidoglycan compositions in control and Fe-depeleted conditons

PG chromatograph, compositions and cross-linkage of WT and Δ*elyC* cells grown with or without 375 μM 2,2'-dipyridyl (Dip). (A) PG profiles of WT and Δ*elyC* mutant strains at 21°C. Representative chromatograms of muramidase-digested PG samples of WT and Δ*elyC* cells at 21°C. (B) Table summarize the relative molar abundance (%) of monomers, dimers, trimers, dipeptide (di), tripeptide (tri) and tetrapeptide (tetra) containing

muropeptides, lipoprotein-bound muropeptides (Lpp) and muropeptides with a residue of (1-6 anhydro) N-acetylmuramic acid (anhydro). Data regarding the % of cross-linkage (proportion of crosslinked peptide side chains, calculated on dimers and trimers content) is also included. (C) Relative molar abundance of the interesting peaks indicated in the chromatograms is calculated from the relative area of every peak and expressed as mean % value from three independent samples. Statistical analysis: t-test comparing each sample to the WT, in each condition. Asterisks indicate significant differences ($P < 0.01$). Culture conditions are indicated in materials and methods. Values represent mean from three independent cultures. M4: [N-acetylglucosamine (NAG)-N-acetylmuramic acid (NAM)]-tetrapeptide; M3: NAG-NAM-tripeptide ; M2: NAG-NAM-dipeptide ; M3-Lpp: NAG-NAM-tripeptide-Braun's lipoprotein (Lpp) ; N denotes termination in an anhydro-residue.

A

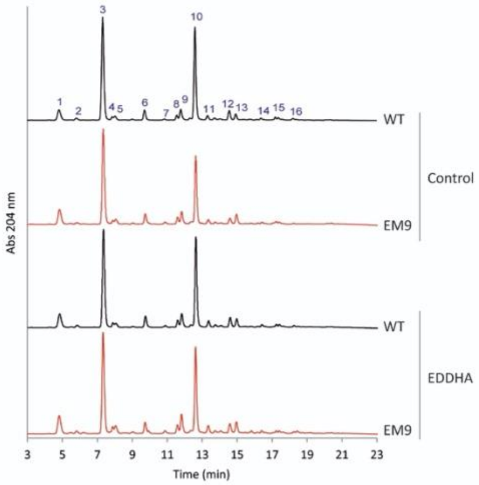

B

Parameter	wt		EM9		wt EDDHA		EM9 EDDHA	
	%	SD	%	SD	%	SD	%	SD
Monomers	69.5	0.3	73.7 *	0.6	68.7	0.5	69.9	3.3
Dimers	29.5	0.3	24.7 *	0.6	29.9	0.5	28.5	2.7
Trimers	1.0	0.0	1.6 *	0.0	1.3	0.1	1.6	0.6
Di	1.0	0.1	1.1	0.1	1.5 *	0.1	2.0	0.9
Tri	12.6	0.5	16.8 *	0.4	15.2 *	0.5	16.7	1.6
Tetra	83.4	0.5	78.4 *	0.3	80.2 *	0.4	75.8 *	1.5
Penta	3.0	0.1	3.7	0.3	3.1	0.1	5.4	2.1
Lpp	7.1	0.3	6.6	0.3	7.5	0.2	6.6	0.3
Anhydro	1.4	0.2	1.0	0.3	1.6	0.1	1.3	0.2
Crosslink	31.5	0.4	27.9 *	0.7	32.6	0.6	31.6	3.8

C

Peak	Muropep.	wt		EM9		wt EDDHA		EM9 EDDHA	
		%	SD	%	SD	%	SD	%	SD
1	M3	7.5	0.5	11.1 *	0.3	9.5 *	0.4	11.4 *	1.4
2	$M4^G$	1.1	0.2	0.7	0.1	0.8	0.5	1.2	0.4
3	M4	53.1	0.3	52.9	1.0	49.9 *	0.3	46.7 *	1.2
4	M2	1.0	0.1	1.1	0.1	1.5 *	0.1	2.0	0.9
5	M5	2.1	0.1	2.7	0.3	1.8	0.1	3.9	2.2
6	M3-Lpp	4.8	0.2	5.2	0.4	5.4	0.2	4.7	0.3
7	D33	0.2	0.0	0.5 *	0.0	0.4 *	0.0	0.6 *	0.1
8	D34	1.0	0.1	1.5 *	0.1	1.4 *	0.1	1.6	0.4
9	D43	2.7	0.0	3.1	0.2	3.2	0.1	4.1	0.9
10	D44	21.1	0.3	16.3 *	0.2	20.0	0.4	17.5 *	1.3
11	D45	0.9	0.1	1.0	0.0	1.3	0.2	1.5 *	0.1
12	D43-Lpp	2.2	0.0	1.4 *	0.1	2.1	0.1	1.9 *	0.0
13	T444	0.9	0.0	1.5 *	0.1	1.1 *	0.0	1.4	0.5
14	D43N	0.3	0.1	0.4	0.1	0.5	0.0	0.4	0.1
15	D44N	0.9	0.1	0.6	0.2	1.0	0.2	0.7	0.0
16	T444N	0.2	0.0	0.1	0.0	0.2	0.1	0.2	0.1

Figure S9. Wild-type and Δ*elyC* cells peptidoglycan compositions in control and EDDHA Fe-depeleted conditons

PG chromatograph, compositions and cross-linkage of WT and Δ*elyC* cells grown with or without 250 μM ethylenediamin’-N,N'-bis(2-hydroxyphenylacetic) acid (EDDHA). (A)

PG profiles of WT and Δ*elyC* mutant strains at 21°C. Representative chromatograms of muramidase-digested PG samples of WT and Δ*elyC* cells at 21°C. (B) Table summarize the relative molar abundance (%) of monomers, dimers, trimers, dipeptide (di), tripeptide (tri) and tetrapeptide (tetra) containing muropeptides, lipoprotein-bound muropeptides (Lpp) and muropeptides with a residue of (1-6 anhydro) N-acetylmuramic acid (anhydro). Data regarding the % of cross-linkage (proportion of crosslinked peptide side chains, calculated on dimers and trimers content) is also included. (E-F) Relative molar abundance of the interesting peaks indicated in the chromatograms is calculated from the relative area of every peak and expressed as mean % value from three independent samples. Statistical analysis: t-test comparing each sample to the WT, in each condition. Asterisks indicate significant differences (*P*< 0.01). Culture conditions are indicated in materials and methods. Values represent mean from three independent cultures. M4: [N-acetylglucosamine (NAG)-N-acetylmuramic acid (NAM)]-tetrapeptide; M3: NAG-NAM-tripeptide ; M2: NAG-NAM-dipeptide ; M3-Lpp: NAG-NAM-tripeptide-Braun's lipoprotein (Lpp) ; N denotes termination in an anhydro-residue.

Table S2. Chromate at 125 µM does not influence LB pH

	Mean ± SD of 3 independent replicates*
LB	6.437 ± 0.031
LB + 125 µM K_2CrO_4	6.440 ± 0.036

* pH were measured with Fisherbrand™ accumet™ AB15 pH meter

SI References:

1. Datsenko KA, Wanner BL. 2000. One-step inactivation of chromosomal genes in *Escherichia coli* K-12 using PCR products. Proc Natl Acad Sci U S A 97:6640-5.
2. Chung CT, Niemela SL, Miller RH. 1989. One-step preparation of competent *Escherichia coli*: transformation and storage of bacterial cells in the same solution. Proc Natl Acad Sci U S A 86:2172-5.

3. Johnson JE, Lackner LL, Hale CA, de Boer PA. 2004. ZipA is required for targeting of DMinC/DicB, but not DMinC/MinD, complexes to septal ring assemblies in *Escherichia coli*. J Bacteriol 186:2418-29.
4. Yu D, Ellis HM, Lee EC, Jenkins NA, Copeland NG, Court DL. 2000. An efficient recombination system for chromosome engineering in *Escherichia coli*. Proc Natl Acad Sci U S A 97:5978-83.
5. Blattner FR, Plunkett G, 3rd, Bloch CA, Perna NT, Burland V, Riley M, Collado-Vides J, Glasner JD, Rode CK, Mayhew GF, Gregor J, Davis NW, Kirkpatrick HA, Goeden MA, Rose DJ, Mau B, Shao Y. 1997. The complete genome sequence of *Escherichia* coli K-12. Science 277:1453-62.
6. Paradis-Bleau C, Kritikos G, Orlova K, Typas A, Bernhardt TG. 2014. A genome-wide screen for bacterial envelope biogenesis mutants identifies a novel factor involved in cell wall precursor metabolism. PLoS Genet 10:e1004056.
